# Poltergeist-Like 2 (PLL2)-dependent activation of herbivore defence distinguishes systemin from other immune signalling pathways

**DOI:** 10.1038/s41477-025-02040-7

**Published:** 2025-07-04

**Authors:** Rong Li, Xu Wang, Fatima Haj Ahmad, Anja Thoe Fuglsang, Anke Steppuhn, Annick Stintzi, Andreas Schaller

**Affiliations:** 1 Department of Plant Physiology and Biochemistry, Institute of Biology, University of Hohenheim, Stuttgart, Germany; 2 Department of Biotechnology, Faculty of Agricultural Technology, Al-Balqa Applied University, Al-Salt, Jordan; 3 Department of Plant and Environmental Sciences, University of Copenhagen, Frederiksberg C, Denmark; 4 Department of Molecular Botany, Institute of Biology, University of Hohenheim, Stuttgart, Germany

**Keywords:** Pattern recognition receptors in plants, Herbivory, Plant signalling, Wounding, Biotic

## Abstract

Systemin, the first signalling peptide identified in plants, mediates induced resistance against insect herbivores and necrotrophic pathogens in tomato^1–3^. Initially, systemin was conceived as a hormone-like, long-distance messenger that triggers systemic defence responses far from the site of insect attack. It was later found to rather act as a phytocytokine, amplifying the local wound response for the production of downstream signals that activate defence gene expression in distant tissues^4^. Systemin perception and signalling rely on the systemin receptor SYR1^5^. However, the specifics of SYR1-dependent signalling and how systemin signalling differs from other immune signalling pathways remain largely unknown. Here we report that systemin activates the poltergeist-like phosphatase PLL2 in a SYR1-dependent manner. PLL2, in turn, regulates early systemin responses at the plasma membrane, including the rapid inhibition of proton pumps through dephosphorylation of their regulatory C-termini. PLL2 was found to be essential for downstream defence gene induction, ultimately contributing to insect resistance.

## Abstract (web summary)

The phosphatase PLL2 was identified as a specific component in systemin-induced immune signalling. PLL2 regulates proton pump activity at the plasma membrane and is required for downstream defence gene induction and insect resistance.

## Main

Like other phytocytokines, the 18-amino-acid systemin peptide is derived from a larger precursor protein by proteolytic processing^6,7^. Rapid cellular responses to systemin include the extracellular alkalinization response, an increase in cytosolic calcium, depolarization of the plasma membrane and the induction of an oxidative burst^5,8–11^. These early systemin responses resemble those triggered by other phytocytokines and are hallmarks of pattern-triggered immune signalling^12–16^. They depend on the systemin receptor SYR1 resembling many other peptide and pattern recognition receptors (PRRs) in the large family of leucine-rich receptor kinases^5^. However, despite apparent similarities in perception mechanism and early signal transduction events, systemin induces distinct downstream responses including the activation of the octadecanoid pathway for the production of jasmonates that are responsible for the systemic induction of defence responses against insects and necrotrophic pathogens, rather than pattern-triggered immunity (PTI)^1,3,17^.

**Fig. 1:**
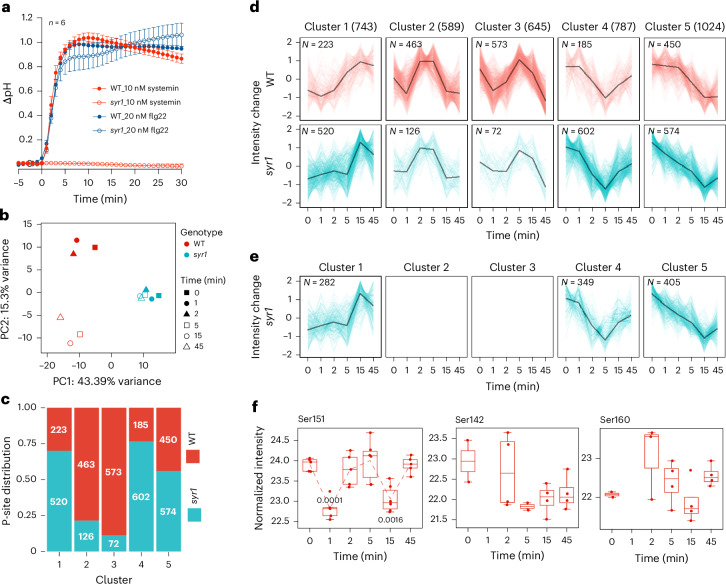
The systemin response is characterized by transient dephosphorylation of cellular proteins at 1 min after treatment. **a**, Extracellular alkalinization is an early hallmark of systemin and pattern-triggered immune signalling. The pH of the culture medium was recorded after addition of 10 nM systemin or 20 nM flg22 to wild-type and *syr1* cells. Data points represent the mean ± standard error (s.e.) of six independent experiments. WT, wild type. **b**, The systemin-induced phospho-proteomic response of wild-type cells is lost in *syr1*. PCA is shown for phospho-site datasets (average of six biological replicates for each genotype and time point after systemin treatment). **c**,**d**, *K*-means clustering of phospho-sites (P-sites) according to their temporal changes in intensity identified five time profiles (clusters) between the wild-type and *syr1* cell cultures (**d**), with the number of sites per genotype and cluster summarized in **c**. A transient drop in intensity is frequently observed in wild type but not in *syr1* (clusters 2 and 3 in **c** and **d**). **e**, Phosphosites that belong to clusters 2 and 3 in wild type distribute to other clusters in *syr1*. The black lines in **d** and **e** show the median of phospho-site intensity for each cluster. **f**, Systemin-induced changes in phosphorylation at Ser151, Ser142 and Ser160 of SlPLL2 in wild-type cells. The experiment included six biological replicates. Boxes range from 25th to 75th percentiles with the splitting line at the median. Whiskers extend to the minimum and maximum values if lower than 1.5× interquartile range. For Ser151, which has data for each time point, medians are connected by a trend line. Adjusted *P* values are shown for time points significantly different from *t* = 0 min (two-tailed ANOVA with Dunnett’s multiple comparison test). Source data

To get insight into the specific signalling events downstream of the SYR1 receptor kinase, we used phospho-proteomics in a systemin-responsive cell culture from a wild tomato species, *Solanum peruvianum*. In a previous study, we had compared the response of these cells to systemin and to an inactive control peptide^18^. Here, focusing on SYR1-dependent signalling events, we compared systemin-induced changes in protein phosphorylation in wild-type and *syr1* mutant cell cultures, generated by biolistic transformation with a clustered regularly interspaced short palindromic repeats and CRISPR-associated protein 9 (CRISPR/Cas9) genome editing construct (Supplementary Table 1). A rapid increase in medium pH, that is, the systemin-induced alkalinization response, was observed for wild type but not for *syr1*. Confirming the specific loss only of *SYR1* function, the response to the flg22 peptide from bacterial flagellin that depends on FLS2 as PRR was unaffected in *syr1* cells (Fig. 1a).

**Extended Data Fig. 1:**
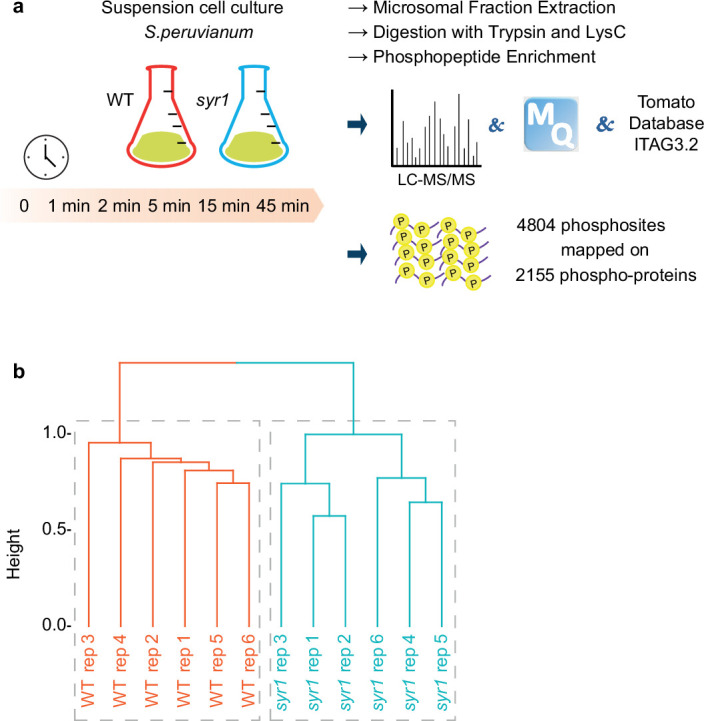
Design of the phospho-proteomics experiment. **a**, Phospho-proteomics workflow. Samples were harvested from WT and *syr1* cell cultures before and at 5 time points after systemin treatment in six biological replicates, resulting in a total of 72 samples. From each sample, the microsomal membrane fraction was extracted and digested with trypsin and LysC. After phospho-peptide enrichment, samples were analyzed by LC-MS/MS. Raw files were transferred to MaxQuant for peptide quantification using the tomato proteome database ITAG3.2 as the reference. **b**, Hierarchical clustering of the six biological replicates clearly separated the two genotypes.

For phospho-proteomics, *syr1* and wild-type cells were collected in a time series after systemin treatment in six biological replicates (Extended Data Fig. 1a,b). Microsomal membranes were isolated from each sample, digested with Lys-C and trypsin, enriched for phosphopeptides and subjected to mass spectrometry (liquid chromatography with tandem mass spectrometry (LC-MS/MS)) in untargeted data-dependent acquisition mode. Quantitative information was obtained for 4,804 phosphopeptide species matching 2,155 different proteins (Supplementary Table 2).

Principal component analysis (PCA) of phosphosites that responded to systemin treatment (sites differentially phosphorylated at *P* < 0.05, comparing all time points to time 0) clearly separated wild-type and *syr1* samples; 43.4% of the total variance is explained by differences between the two genotypes (Fig. 1b). The second principal component separated wild type according to sampling time reflecting systemin-induced changes in protein phosphorylation. By contrast, all *syr1* samples clustered closely together indicating that there is no such systemin response in the receptor mutant (Fig. 1b).

**Extended Data Fig. 2:**
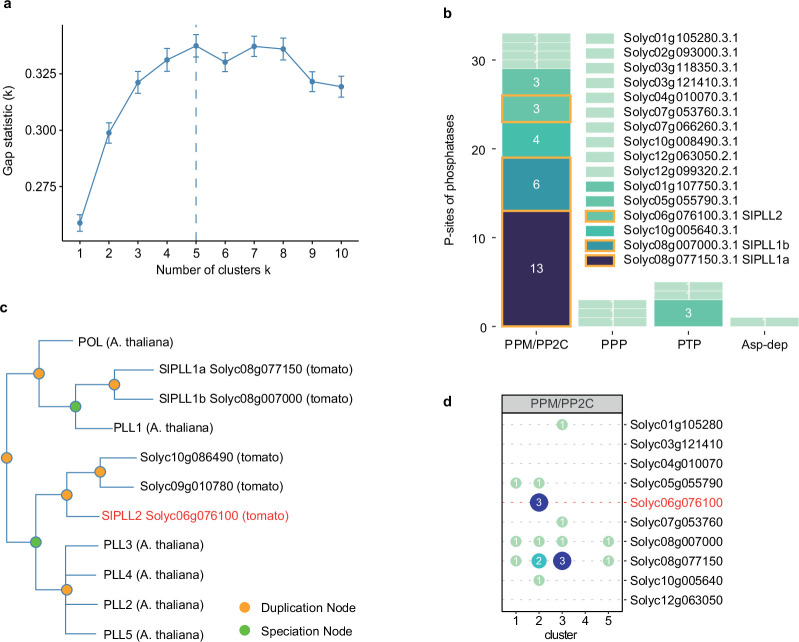
Selection of PLL2 as a candidate phosphatase specifically involved in systemin signalling. **a**, The K-means clustering algorithm requires a predefined number of clusters K that is chosen to best describe the structure of the data. Gap statistics showed that the cluster structure showed maximum distance from the random uniform distribution of points with the optimal number of K = 5 clusters. 10 was set as the upper limit for K and 100 bootstrapping runs were performed in fviz_nbclust^52^ (method = “gap_stat”). Data points show the mean of 100 bootstrapping runs ± SE. **b**, Protein phosphatases represented in this phosphoproteome dataset belonged to four different families: metallo-dependent protein phosphatases (PPM)/ type 2C protein phosphatases (PP2C), phosphoprotein phosphatases (PPP), protein tyrosine phosphatases (PTP) and aspartate (Asp)-dependent phosphatases. Intensity of shading is used to indicate the number of detected phospho-sites with darker colors indicating more phospho-sites for a given protein. Most phospho-sites were found in POLTERGEIST-LIKE phosphatases (POL, boxed in orange). **c**, Phylogenetic tree of POL family proteins from Arabidopsis and tomato. The tree was generated by PhyloGenes (v4.1) based on GIGA algorithm^62^. SlPLL2 (highlighted in red) is located in a sister clade to Arabidopsis PLL2 – PLL5. **d**, SlPLL2 has three phospho-sites that show a transient drop in intensity at 1 min after systemin treatment (cluster 2). Cluster distribution is shown for the phospho-sites of PPM/PP2C family phosphatases. The number of phospho-sites in each cluster is reflected by the intensity of color shading and size of the dots. SlPLL2 (Solyc06g076100) stands out as all three differentially phosphorylated sites belong to cluster 2.

We then performed *k*-means clustering of phospho-peptide abundance profiles to compare dynamic changes in protein phosphorylation between the two cell cultures (Fig. 1d, Extended Data Fig. 2a and Supplementary Table 2). The largest difference between wild type and *syr1* was observed for clusters 2 and 3, characterized by a rapid and transient decrease in phosphorylation at 1 min after systemin treatment. Less than 20% (198) of the peptides in these clusters were from *syr1*, compared to >80% (1,036) from the systemin-responsive wild type (Fig. 1c). We then checked whether these 1,036 peptides show time-dependent changes in phosphorylation in *syr1* cells and, if so, to which cluster they belong. We found them in all clusters except 2 and 3; none of them showed the systemin-induced drop in abundance at 1 min after treatment (Fig. 1e). The data indicate that the SYR1-mediated systemin response is characterized by rapid and transient dephosphorylation of cellular proteins, implying the activation of protein phosphatases early in systemin signalling.

Most of the protein phosphatases showing SYR1-dependent changes in phosphorylation belonged to the metallo-dependent protein phosphatase (PPM)/type 2C protein phosphatase (PP2C) family, and the largest number of phosphosites differentially regulated after systemin treatment was observed for poltergeist-like phosphatases (PLLs; Extended Data Fig. 2b,c), representing clade C in the PP2C family^19^. We focused our attention on PLL2 (Solyc06g076100) for which all three detected phosphosites showed a cluster 2-type response to systemin treatment (Extended Data Fig. 2d). PLL2 was transiently dephosphorylated in its N-terminal domain at Ser151, and likely also at Ser142 and Ser160 (Fig. 1f). The latter two sites were undetectable at 1 min after systemin treatment, consistent with a decrease in abundance due to dephosphorylation. However, as these phosphopeptides were not reliably detected in all biological replicates, with some data points missing also at other time points (Supplementary Note), the evidence for dephosphorylation is not as strong as for the Ser151 phosphosite.

**Fig. 2:**
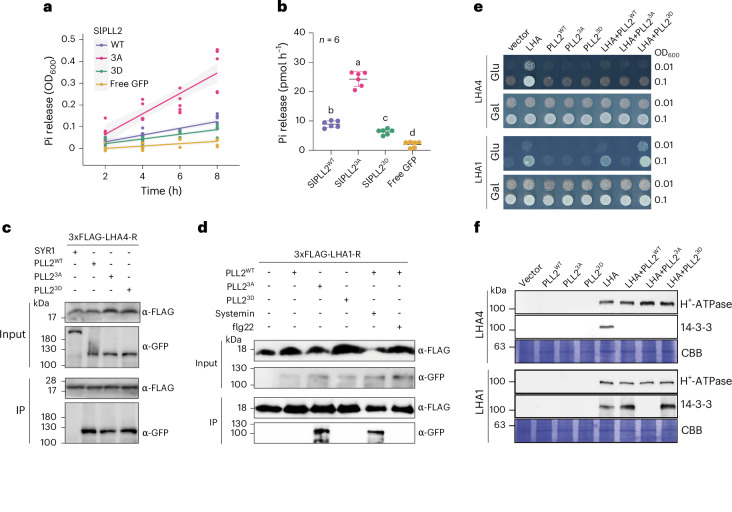
The dephosphorylated, active form of SlPLL2 inhibits tomato P-type H^+^-ATPases. **a**,**b**, SlPLL2 activity depends on its phosphorylation status. Equal amounts of SlPLL2^WT^, phospho-dead SlPLL2^3A^, phospho-mimetic SlPLL2^3D^ and free GFP (negative control) purified from agro-infiltrated *N. benthamiana* leaves (Extended Data Fig. 3) were used in activity assays based on the colorimetric quantification of inorganic phosphate (Pi) release from a synthetic phospho-peptide substrate (pLHA1, 100 µM). Data represent the mean ± s.d. of six independent experiments. Different letters indicate significant differences at *P* < 0.05 (two-tailed ANOVA followed by Tukey test). OD_600_, optical density at 600 nm. **a**, Progress curves of phosphate release. **b**, Activity of SlPLL2 variants in pmol phosphate per hour. **c**,**d**, Co-IP assay of SlPLL2 interaction with LHA1 and LHA4. SlPLL2 phospho-variants were co-expressed as GFP fusions with the Flag-tagged regulatory (R) domain of LHA4 (**c**) or LHA1 (**d**) in *N. benthamiana* leaves. Total protein extracts from leaves treated with 100 nM systemin or flg22 were immunoprecipitated with anti-Flag agarose beads, then detected with anti-GFP on western blots. Co-IP assays were performed in triplicate for **c** and in duplicate for **d**. **e**, Growth assays showing SlPLL2-mediated regulation of H^+^-ATPase activity in yeast. Yeast strain RS-72 was (co-)transformed with expressions constructs for tomato H^+^-ATPases LHA1 (two lower panels) or LHA4 (two upper panels) and SlPLL2 phospho-variants in different combinations. Cells were spotted in two dilutions (OD_600_ = 0.1 or 0.01) on control medium (Gal) and on selective medium (Glu), where growth depends on LHA1 or LHA4 activity. Results are representative of three biological replicates. **f**, Western blot overlay monitoring the phosphorylation status of the regulatory threonine of LHA1 and LHA4. Microsomal fractions (100 µg) of yeast cultures (co-)expressing LHA1 (lower panel) or LHA4 (upper panel) with SlPLL2 variants were separated by SDS–PAGE and analysed on western blots using an antiserum against the *Arabidopsis* H^+^-ATPase (AHA2); 14–3–3 protein binding distinguishes the penultimate threonine in its phosphorylated state from the non-phosphorylated state. Coomassie brilliant blue-stained gels (CBB) are shown as loading control. Experiments were performed three times with similar results. In **e** and **f**, equal expression of PLL2 variants in yeast was confirmed by western blot analysis (Supplementary Fig. 1). Source data

**Extended Data Fig. 3:**
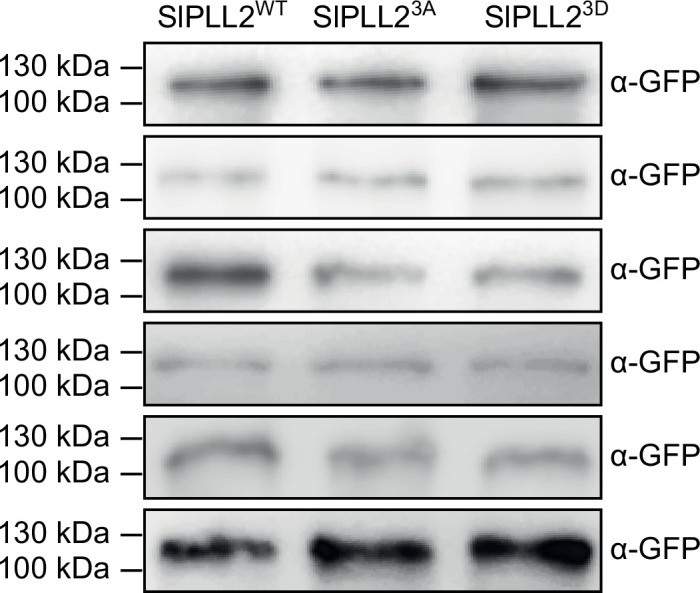
Quantification of SlPLL2 variants in protein extracts used for phosphatase activity assays. As shown in Fig. 2a,b. SlPLL2^WT^, SlPLL2^3A^ and SlPLL2^3D^ were expressed as C-terminal GFP fusion proteins in *N. benthamiana*, and purified from leaf extracts by GFP-trap (Chromotek). Abundance of the GFP fusion proteins was analyzed by anti-GFP immunoblotting. In each of the six independent experiments, similar expression levels were observed for the three SlPLL2 variants. Source data

Addressing the effect of reversible phosphorylation on PLL2 activity, we performed site-directed mutagenesis to replace all three serines with phospho-mimetic aspartate or non-phosphorylatable (phospho-dead) alanine residues. The wild-type enzyme (PLL2^WT^) as well as its phospho-mimic (PLL2^3D^) and phospho-dead (PLL2^3A^) variants were expressed in *Nicotiana benthamiana* leaves as green fluorescent protein (GFP) fusions and purified by immuno-precipitation (GFP-trap; Extended Data Fig. 3). Phosphatase activity of the recombinant proteins was analysed in vitro as the amount of inorganic phosphate released from a synthetic phosphopeptide substrate. PLL2^3A^ showed threefold higher phosphatase activity, while the phospho-mimic PLL2^3D^ mutant was significantly less active compared to the wild-type enzyme (Fig. 2a,b). The data indicate that PLL2 is activated by dephosphorylation in response to systemin in a SYR1-dependent manner. Activated PLL2 may regulate downstream proteins by dephosphorylation, thereby contributing to the transient drop in phosphosite abundance observed after systemin treatment.

**Extended Data Fig. 4:**
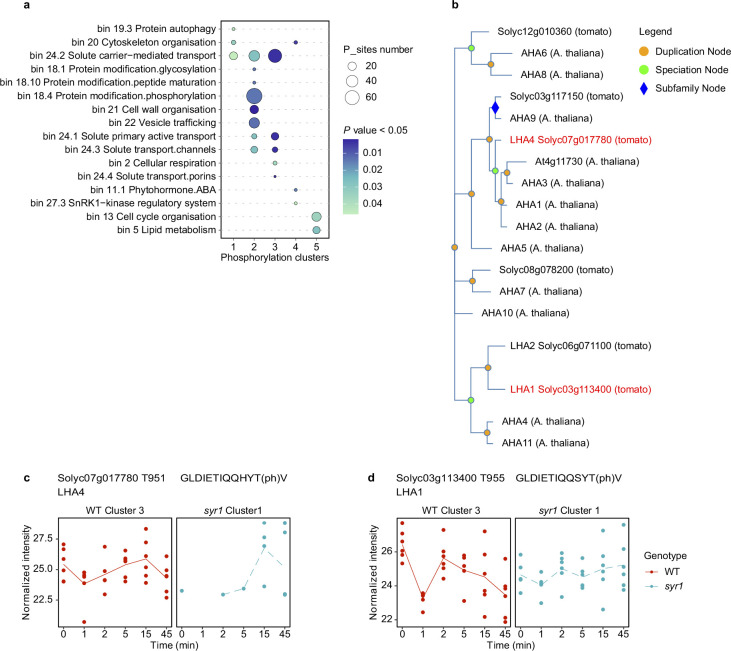
Identification of plasma membrane proton pumps LHA1 and LHA4 as candidate substrates of SlPLL2. **a**, over-representation analysis of biological processes in the five clusters. The proteins represented by the phospho-sites in each of the five clusters were subjected to MapMan over-representation analysis. MapMan bins of biological processes that were significantly (*P* < 0.05) enriched are plotted according to cluster number. The size of the dots reflects the number of phospho-sites for each bin, color shading indicates *P* value (hypergeometric test, one-sided). Bins related to solute transport (bin 24) are enriched in clusters 2 and 3 including LHA1 and LHA4. **b**, phylogenetic analysis of tomato and Arabidopsis P-type H^+^-ATPases. The tree was generated by PhyloGenes (v4.1) based on GIGA algorithm^62^. LHA1 and LHA4 are highlighted in red. **c**, **d** normalized intensity of phosphorylation of the regulatory threonine T951 of LHA4 (**c**, Solyc07g017780) and T955 of LHA1 (**d**, Solyc03g113400) is shown in a time series after systemin treatment of the *S. peruvianum* wild-type (WT; red) and *syr1* (blue) cell cultures in six biological replicates. The trendline connects the mean intensity at each time point.

MapMan (BinTree version X4 Release 2.0) was used to functionally characterize potential PLL2 targets among cluster 2 and 3 proteins showing the systemin-induced dephosphorylation response. Among the MapMan bins significantly enriched in clusters 2 and 3 are ‘solute carrier-mediated transport’ (bin 24.2) and ‘solute primary active transport’ (bin 24.1) (Extended Data Fig. 4a), including two plasma membrane proton pumps, LHA1 (Solyc03g113400) and LHA4 (Solyc07g017780), as putative targets of PLL2 (Extended Data Fig. 4b). LHA1 was also suggested as a putative PLL target in our previous study comparing phosphoproteomic responses to systemin and an inactive control peptide^18^. Here we observed the dephosphorylation of LHA1 and a second plasma membrane proton pump (LHA4) at 1 min after systemin treatment. Transient dephosphorylation was more pronounced for LHA1 compared to LHA4, and it depended on SYR1 (Extended Data Fig. 4c,d), suggesting a role for LHA1 downstream of SYR1 in the systemin signalling pathway.

Plasma membrane H^+^-ATPases have previously been implicated in systemin signalling in tomato, and in wound signalling in general^20–22^. Reduced proton extrusion as a result of proton pump inhibition may contribute to extracellular alkalinization and membrane depolarization, which are both necessary and sufficient for the activation of downstream defence gene expression^20,23,24^. The activity of plasma membrane H^+^-ATPases is controlled by reversible phosphorylation, with the second-to-last C-terminal threonine residue as the main regulatory site^25,26^. Phosphorylation of this residue and subsequent binding of 14–3–3 proteins activates the proton pump, while dephosphorylation reduces proton pump activity^27,28^. This regulatory threonine is the site that was found to be dephosphorylated in LHA1 and LHA4 in response to systemin treatment (Extended Data Fig. 4c,d). This is also the site that was dephosphorylated by PLL2 in the synthetic phospho-peptide pLHA1 that was used as the substrate in our in vitro activity assay (Fig. 2a,b). The results therefore indicated that PLL2 is able to dephosphorylate the regulatory phospho-threonine from the C-terminus of LHA1, with highest activity for the phospho-dead PLL2^3A^ mutant (Fig. 2a,b). The data suggest that systemin-induced dephosphorylation and activation of PLL2 contribute to the alkalinization response by inhibition of plasma membrane proton pump activity.

**Extended Data Fig. 5:**
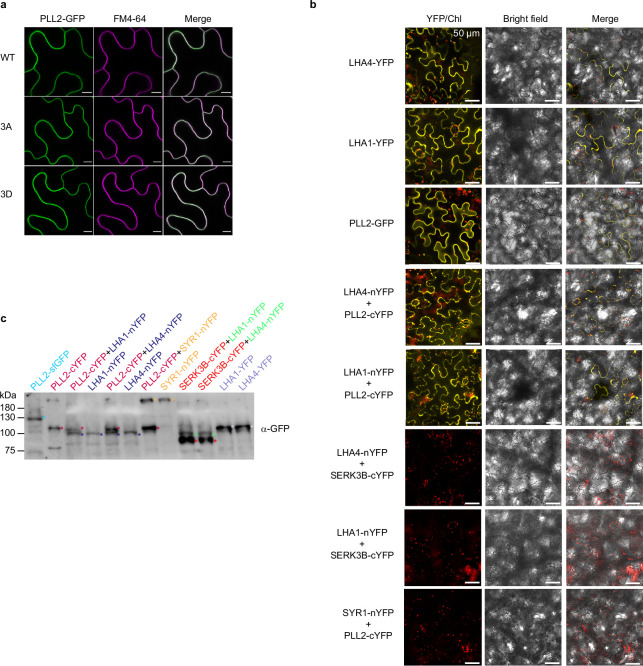
LHA1/4 and SlPLL2 interact at the plasma membrane. **a**, SlPLL2 localizes to the plasma membrane irrespective of the phosphorylation status of S142-S151-S160. SlPLL2-GFP (WT, 3A and 3D mutants) were transiently expressed in *N. benthamiana* leaves. Leaf pieces were incubated in 10 µM FM4-64 (plasma membrane marker) 15 min before confocal imaging using a Zeiss LSM980 microscope in the red (FM4-64) and green (GFP) channels, respectively; scale bar = 10 µm. **b**, Interaction of LHA1/4 and SlPLL2 is indicated by bimolecular fluorescence complementation (BiFC). Tomato H^+^-ATPases (LHA1 and LHA4) fused with nYFP, and SlPLL2 with cYFP were transiently expressed in *N. benthamiana* leaves. LHA1 and LHA4 fused with full-length YFP, and SlPLL2 with GFP were used as positive controls. Negative controls included leaves expressing LHA1-nYFP or LHA4-nYFP together with SERK3B-cYFP, and SlPLL2-cYFP co-expressed with SYR1-nYFP; scale bar = 50 μm. **a**, and **b**, the expriments were repeated at least three times with similar results. **c**, All fusion proteins used in the BiFC experiment are expressed in *N. benthamiana* leaves. Anti-GFP western blot analysis of protein extracts from *N. benthamina* leaves used in the BiFC experiment shown in (**b**). Colored asterisks mark the bands of the corresponding fusion proteins. This control blot was done for one representative experiment.

To corroborate this hypothesis, we first tested whether PLL2 co-localizes with the proton pumps at the plasma membrane. A potential myristoylation site for plasma membrane targeting is tentatively predicted for PLL2 (ref. ^29^). Plasma membrane localization was indeed observed for PLL2^WT^ and also for the PLL2^3A^ and PLL2^3D^ variants transiently expressed in *N. benthamiana* leaves (Extended Data Fig. 5a). Interaction of PLL2 with the plasma membrane H^+^-ATPases LHA1 and LHA4 was confirmed in *N. benthamiana* by bimolecular fluorescence complementation (BiFC; Extended Data Fig. 5b,c) and in co-immunoprecipitation (Co-IP) experiments testing for pull-down of GFP-tagged PLL2 by the flag-tagged regulatory (R) domain of the proton pumps. This experiment confirmed interaction of PLL2 with LHA4, which was observed for all three PLL2 variants (Fig. 2c). Hence, the interaction with LHA4 does not seem to depend on the phosphorylation status of PLL2. By contrast, only the hyperactive PLL2^3A^ mutant was found to interact with the regulatory C-terminus of LHA1 (LHA1-R; Fig. 2d). For PLL2^WT^, interaction with LHA1-R was observed only after systemin treatment, which is consistent with the systemin-induced dephosphorylation and activation of PLL2 (Fig. 2d). No interaction was observed in response to flg22 (Fig. 2d). The data indicate that PLL2 needs to be in its dephosphorylated/activated state to interact with LHA1, and they further suggest that the SYR1-dependent dephosphorylation and activation of PLL2 distinguishes systemin from flg22-induced signalling.

To test whether PLL2 controls proton pump activity in vivo, we first used the yeast strain RS-72 which expresses the endogenous H^+^-ATPase, *PMA1*, under the control of the *GAL1* promoter and, therefore, requires galactose as the sole carbon source for growth. On media containing glucose, the endogenous proton pump is not expressed, and growth depends on the activity of plasmid-borne plant H^+^-ATPases^30^. Both LHA4 and LHA1 supported yeast growth indicating that the two pumps are expressed in yeast and active (Fig. 2e). Binding of 14–3–3 proteins in a western blot overlay confirmed the presence of the activating phospho-threonine at the regulatory C-termini of the proton pumps (Fig. 2f). Yeast growth and phosphorylation of LHA1 were suppressed upon co-expression of PLL2, and this inhibition depended on PLL2 activity: it was strongest for the hyperactive PLL2^3A^ mutant, weaker for PLL2^WT^ and not observed for PLL2^3D^ (Fig. 2e,f, bottom panels). The data support our hypothesis that the alkalinization response depends on the systemin-mediated dephosphorylation and activation of PLL2 which, in turn, dephosphorylates and thereby inactivates the proton pump LHA1. PLL2-mediated inhibition of LHA4, however, did not seem to depend on the phosphorylation status of PLL2. Yeast growth was impaired, and phosphorylation of LHA4 was inhibited by co-expression of all three PLL2 variants (Fig. 2e,f, upper panels). This result is consistent with our Co-IP experiments, which showed interaction of the regulatory LHA4 C-terminus with wild-type PLL2 as well as the PLL2^3A^ and PLL2^3D^ mutants (Fig. 2c). Apparently, all three PLL2 variants are able to bind LHA4, irrespective of phosphorylation status, thereby preventing proton pump phosphorylation and activation, resulting in impaired yeast growth.

**Fig. 3:**
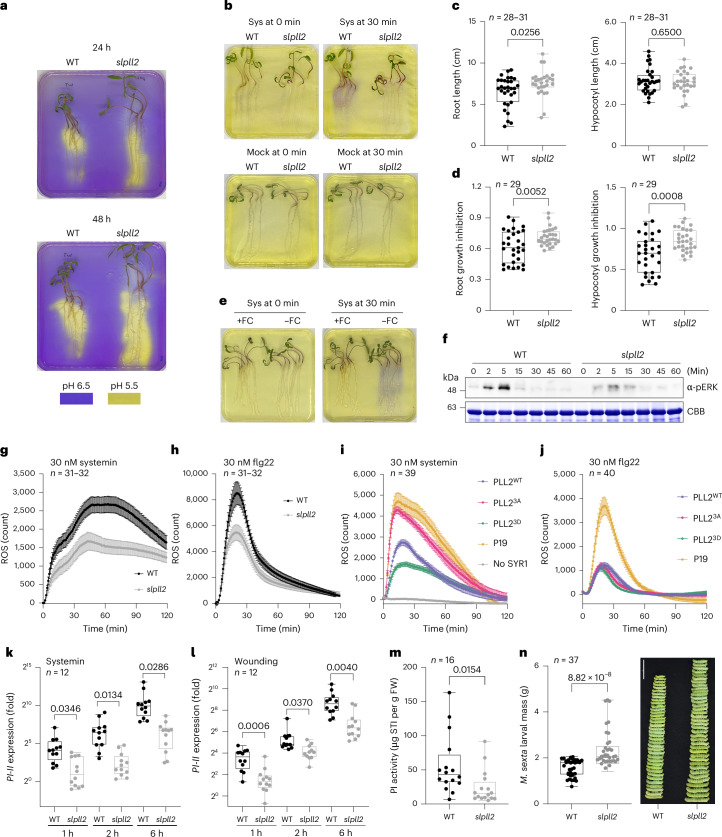
Systemin responses depend on SlPLL2. **a**, Loss of PLL2 function leads to increased proton extrusion in *pll2* compared to wild-type seedlings. Extracellular pH was monitored in the rhizosphere of seedlings transferred from ATS medium^35^ to bromocresol purple (BCP) pH indicator plates at pH 6.5. Changes in medium colour were recorded after 24 and 48 h. **b**, Systemin-induced increase in extracellular pH depends on PLL2. After transfer to BCP indicator plates at pH 5.5, seedlings were sprayed with systemin (1 μM) or water (mock). Medium colour was recorded at 0 and 30 min after spraying. Sys, systemin. In **a** and **b**, similar results were obtained in three independent experiments. **c**, Root growth is enhanced in *pll2* compared to wild-type seedlings grown on ATS medium (*n* = 31 for wild type and 28 for *pll2*). **d**, Systemin-induced growth inhibition is impaired in *pll2*. Root and hypocotyl growth inhibition in *pll2* and wild-type seedlings is shown as the length of systemin-treated seedlings divided by the average length on control plates (*n* = 29 seedlings of each genotype). In **c** and **d**, two-tailed Student’s *t*-test was used to test for statistically significant differences; *P* values are indicated. The experiments were repeated three times with similar results. **e**, Systemin-induced increase in extracellular pH depends on proton pump inactivation. Wild-type seedlings grown on ATS medium were sprayed with 10 µM fusicoccin (+FC) or water (−FC). After 15 min, seedlings were transferred to BCP indicator plates at pH 5.5 and sprayed with systemin (1 µM). Medium colour was recorded at 0 and 30 min after systemin treatment. Similar results were obtained in three independent experiments. **f**, Systemin-triggered MAPK activation is reduced in *pll2*. Extracts (30 µg of protein) of systemin (1 μM)-treated plants were analysed by immunoblotting using an anti-pERK antibody. A Coomassie brilliant blue-stained gel is shown as loading control (CBB). Representative results are shown for one of two independent experiments. **g**,**h**, ROS production is impaired in *pll2*. ROS production was analysed in leaf discs of wild-type and *pll2* plants treated with 30 nM systemin (**g**) or flg22 (**h**). Progress curves show the mean ± s.e. of 31 (*pll2*) and 32 (wild-type) plants. Results are representative for three independent experiments with three different *pll2* alleles (Extended Data Fig. 6). **i**,**j**, The systemin-induced ROS burst correlates with PLL2 activity. The ROS burst was induced by adding 30 nM systemin (**i**) or flg22 (**j**) to leaf discs of *N. benthamiana* plants expressing Spot-tagged SlPLL2^WT^, SlPLL2^3A^ and SlPLL2^3D^ variants or only the P19 vector as control. SYR1-eGFP was co-expressed in **i** to enable systemin perception in *N. benthamiana*. Equal expression of SYR1-eGFP in the presence of different SlPLL2 variants was confirmed by western blot analysis (Supplementary Fig. 2). Data represent the mean ± s.e. of *n* = 39 (in **i**) or 40 (in **j**) plants. The experiment was repeated using GFP-tagged SlPLL2 variants with similar results. **k**,**l**, Induction of *PI-II* expression by systemin and wounding is impaired in *pll2* compared to wild-type plants. RT-qPCR was performed with RNA extracted from leaf tissue collected before and 1, 3 and 6 h after systemin (1 µM) treatment (**k**) or wounding (**l**). Relative *PI-II* expression is shown as fold change over the untreated control (*n* = 12 biological replicates; two-tailed Student’s *t*-test; *P* values are indicated). **m**,**n**, Herbivore defence is impaired in *pll2* compared to wild-type plants. Freshly hatched *M. sexta* were placed on the second oldest leaf of 4-week-old plants and, when all leaf material had been consumed, transferred to progressively younger leaves. **m**, After 5 days, proteinase inhibitor (PI) activity was analysed in systemic (unwounded) leaves. PI activity is shown as soybean trypsin inhibitor (STI) equivalents per gram fresh weight (FW) for *n* = 16 plants. **n**, Mass of *n* = 37 *M. sexta* larvae after 2 weeks of feeding. Scale bar, 5 cm. The experiment was repeated twice with two independent *pll2* lines, showing similar results. In **m** and **n**, two-tailed Student’s *t*-test was used for pairwise comparisons; *P* values are indicated. Box plots in **c**, **d** and **k**–**n** range from the 25th to 75th percentiles with the splitting line at the median. Whiskers extend to the minimum and maximum values if lower than 1.5 × interquartile range. Source data

**Extended Data Fig. 6:**
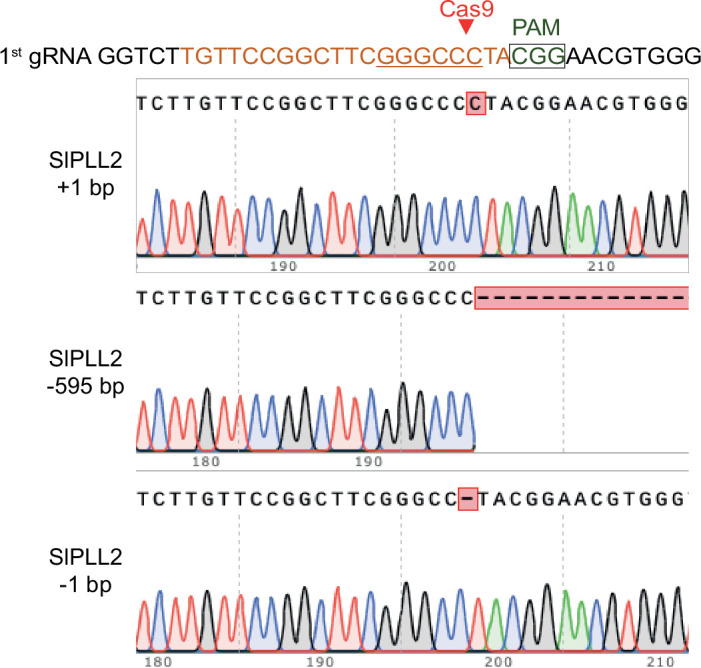
Three independent *pll2* alleles were used in this study. Sequence analysis revealed three independent CRISPR/Cas9-generated mutations at the 1st guide RNA resulting in the addition of one, and deletion of 1 or 595 base pairs (boxed in red) from the *SlPLL2* locus. These mutations resulted in truncated proteins of 145, 147, and 219 amino acids, respectively. The PAM sequence is indicated in green; the Cas9 cleavage site is located 3 bp upstream of the PAM sequence (red arrow).

Loss-of-function analysis, using tomato *pll2* mutants generated by CRISPR/Cas9 genome editing (Extended Data Fig. 6) in root growth assays, corroborated PLL2-mediated regulation of proton pump activity and extracellular pH in planta. As a readout for proton pump activity, we grew seedlings on pH indicator plates and monitored the acidification of the growth medium. Medium acidification was stronger for *pll2* than for wild-type seedlings (Fig. 3a). Consistent with enhanced proton pump activity and the ‘acid growth’ hypothesis^31^, root growth was increased in *pll2* compared to wild type (Fig. 3c). The same assays were also used to test in planta, whether PLL2 is required for systemin-mediated inhibition of proton pump activity and seedling growth. In wild-type seedlings, systemin treatment resulted in extracellular alkalinization, and this response was lost in *pll2* (Fig. 3b). Consistent with increased cell wall pH, seedling growth was inhibited by systemin treatment in both roots and hypocotyls. Growth inhibition was substantially reduced in *pll2* compared to wild-type seedlings (Fig. 3d). The data confirm that systemin-induced pH responses depend on PLL2, possibly by regulation of proton pump activity. Consistent with this notion, systemin-induced pH responses were found to be suppressed by fusicoccin, which stabilizes the complex of H^+^-ATPases with 14–3–3 proteins thereby locking the proton pump in its phosphorylated and activated state. After treatment with fusicoccin, systemin-induced and PLL2-dependent alkalinization of the growth medium were no longer observed (Fig. 3e). The cumulative evidence supports our hypothesis that systemin signalling involves the SYR1-dependent activation of PLL2 which, in turn, dephosphorylates and thereby inactivates the proton pump LHA1 to result in the systemin-induced increase in extracellular pH and growth inhibition.

**Extended Data Fig. 7:**
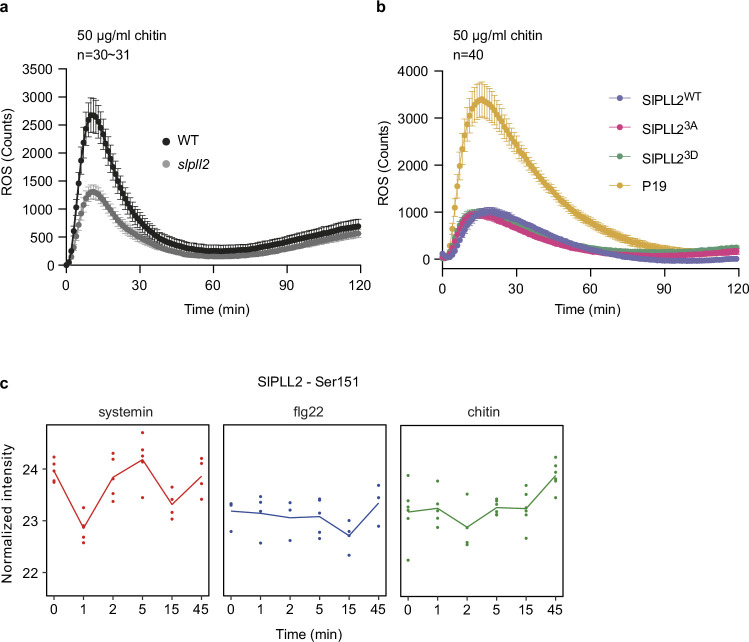
Regulation of PLL2 by dephosphorylation is only observed after systemin treatment and is not involved in chitin signalling. **a**, Chitin-induced ROS production is impaired in *pll2*. ROS production was analyzed in leaf discs of 4-week-old tomato WT (n = 31) and *pll2* (n = 30) plants treated with 50 µg/ml chitin. Progress curves show the mean ± SE. **b**, Tomato PLL2 interferes with chitin-induced ROS production in *N. benthamiana* irrespective of its activity. The ROS burst was analyzed in leaf discs of *N. benthamiana* plants expressing SlPLL2^WT^, SlPLL2^3A^ and SlPLL2^3D^ variants, or the P19 vector only as a control. Data represent the mean ± SE of n = 40 plants for each expression construct. **c**, Rapid and transient dephosphorylation of Ser151 is systemin specific and not observed in response to flg22 or chitin. Normalized intensity of the phospho-peptide comprising the Ser151 phospho-site is shown in a time series after elicitor treatment of the *S. peruvianum* cell culture in six biological replicates. The trendline connects the mean intensity at each time point. Source data

We then tested whether PLL2 function is also required for downstream signalling and systemin-induced defence responses. The oxidative burst observed in wild-type plants in response to systemin treatment was clearly reduced in the *pll2* mutant (Fig. 3g). A similar reduction in reactive oxygen species (ROS) production was also observed after treatment with flg22 or chitin (Fig. 3h and Extended Data Fig. 7a), suggesting that PLL2 is generally required for maximum ROS production after elicitor treatment. Upon overexpression in *N. benthamiana*, tomato PLL2 appeared to interfere with endogenous signalling and attenuated the ROS burst induced by all three elicitors (Fig. 3i,j and Extended Data Fig. 7b). Systemin-induced ROS production was further reduced by the impaired PLL2^3D^ variant and restored to control levels by hyperactive PLL2^3A^ (Fig. 3i). The intensity of the systemin-induced ROS burst thus correlated with PLL2 activity. Interestingly, no such effect of the tomato PLL2 variants was observed on flg22 and chitin-induced ROS production (Fig. 3j and Extended Data Fig. 7b) suggesting that the regulation of PLL2 activity by dephosphorylation of Ser151 and, possibly, serines 142 and 160, is a specific feature of systemin signalling. Supporting this notion, the systemin-induced transient dephosphorylation of Ser151 was not observed after flg22 or chitin treatment (Extended Data Fig. 7c).

**Extended Data Fig. 8:**
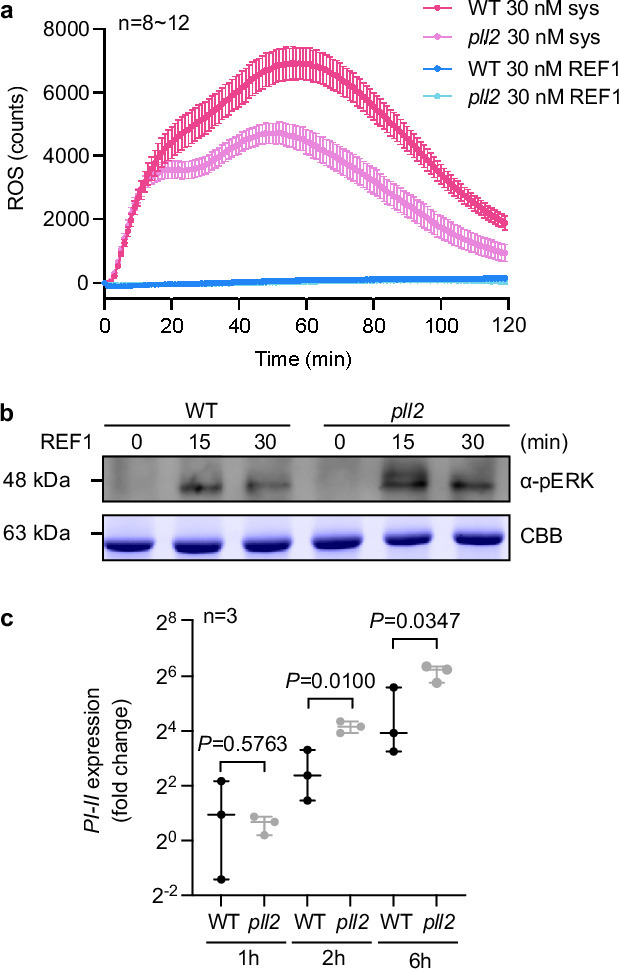
REF1 responses do not depend on PLL2. **a**, there is no ROS burst in response to REF1 treatment. ROS production was analyzed in leaf discs of 4-week-old tomato WT and *pll2* plants treated with 30 nM systemin (n = 12) or REF1 (n = 8). Progress curves show the mean ± SE. **b**, REF1-triggered MAPK activation is not impaired in *pll2*. Leaf tissue was harvested from three-week-old plants in a time series after REF1 (1 μM) treatment. Extracts (30 µg of protein) were analyzed by immunoblotting using an anti-pERK antibody. A Coomassie Brilliant Blue-stained gel is shown as loading control (CBB). Representative results are shown for one of two independent experiments **c**, *PI-II* expression is induced by REF1 to somewhat higher levels in *pll2* compared to WT. Leaf tissue of three-week-old plants was harvested at 1, 2, and 6 h after REF1 (1 µM) treatment for RNA extraction and RT-qPCR analysis. Relative *PI-II* expression is shown as fold change over the untreated control. Results are shown as box plots for n = 3 biological replicates; whiskers extend from the median to maximum and minimum values. Two-tailed Student’s *t*-test was used to compare induction levels between WT and *pll2*; *P* values are indicated). Source data

In addition to the oxidative burst, the activation of mitogen-activated protein kinases (MAPKs) and wounding and systemin-induced defence gene (*PI-II*) expression were also compromised in *pll2* compared to wild type (Fig. 3f,k,l). Consistent with a recent report showing that wound responses mediated by REF1 depend on the PORK1 receptor and are independent of systemin signalling^32^, we found PLL2 not to be required for REF1-mediated defence gene induction (Extended Data Fig. 8). These preliminary data further support a specific role for PLL2 in SYR1-dependent systemin responses. As a result of impaired systemin signalling, *pll2* accumulated lower amounts of defensive proteinase inhibitors than wild-type plants when fed upon by larvae of the specialist herbivore *Manduca sexta* (Fig. 3m). Consequently, larvae gained weight more rapidly on *pll2* mutants compared to wild type (Fig. 3n) indicating a loss of insect resistance. Whether impaired ROS burst, MAPK activation and defence are all consequences of the primary effect of PLL2 on proton pump-mediated pH control, or whether they involve additional PLL2 targets possibly including the SYR2 receptor which was shown to attenuate SYR1 signalling^33^, remains to be investigated.

**Extended Data Fig. 9:**
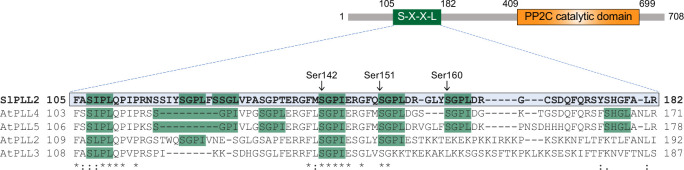
Domain structure of PLL2 and conserved phosphorylation sites in its regulatory N-terminus. Regulatory phosphorylation sites are mostly conserved between tomato PLL2 and Arabidopsis PLL4/5 but differ in function. The sequence alignment shows seven predicted phosphorylation sites in the SXXL/I sequence context (green) in the N-terminal regulatory domain of PLLs. Six of these sites (mutated en block) were shown to be important for the AtPLL4/5 mediated inhibition of pattern-triggered immunity^34^. At least one of these sites, Ser151, and possibly serines 142 and 160 of SlPLL2, were found here to regulate phosphatase activity for the activation of herbivore defence signalling.

In this study, we identified PLL2 as an element of the SYR1-dependent wound signalling pathway for induced herbivore defence. SYR1-dependent activation of PLL2 by dephosphorylation of its regulatory N-terminus is required for the systemin-induced alkalinization response, ROS burst and MAPK activation. These early cellular systemin responses are also hallmarks of PTI. Interestingly and in contrast to tomato PLL2, close homologues in *Arabidopsis* (AtPLL4 and AtPLL5) and rice (XB15) have previously been identified as negative regulators of PTI^34,35^. These PLL2 homologues interact with multiple PRRs to dampen PTI. Upon ligand perception, AtPLL4 and AtPLL5 are phosphorylated at some of the seven predicted phosphorylation sites in their regulatory N-termini (Extended Data Fig. 9) and dissociate from the receptor complex. Thereby inhibition is released and PTI activated^34^. We conclude that closely related PLLs act downstream of SYR1 as well as PRRs but are differentially regulated after perception of the respective ligands. Dephosphorylation and activation of PLL2 is observed specifically in response to systemin and may explain the systemin-specific induction of jasmonate-dependent defence against herbivores and necrotrophic pathogens, while the activation of PTI involves the phosphorylation of AtPLL4/5 to release PRRs from AtPLL4/5-mediated inhibition. The upstream factors controlling the phosphorylation status of PLL2, possibly involving co-receptors of the Somatic Embryogenesis Receptor Kinase family and the receptor-like cytoplasmic kinase Tomato Protein Kinase 1b^11^, remain to be identified.

We further report that activation of PLL2 leads to the dephosphorylation and inhibition of plasma membrane H^+^-ATPases resulting in the rapid increase in extracellular pH after systemin treatment. Later phases of the alkalinization response may not depend on the phosphorylation status and activity of PLL2, because (1) proton pumps are known to be regulated by multiple factors, and (2) other transporters and ion channels also contribute to extracellular pH regulation. PLL2 targets the penultimate phospho-threonine residue in the regulatory C-terminal domain of plasma membrane proton pumps LHA1 and LHA4. The same site is also targeted by auxin signalling. However, in contrast to PLL2-mediated proton pump inhibition and extracellular alkalinization, auxin involves a different phosphatase (PP2C-D) and a pair of antagonistic kinases (TMK1 and TMK4) to increase phosphorylation of the regulatory threonine, thereby stimulating proton extrusion and acid growth^36–39^. In addition to the penultimate phospho-threonine residue, there are multiple other phosphosites in the regulatory C-terminus of the proton pump. Flg22-induced proton pump inhibition is achieved by the dephosphorylation of two activating sites (Thr881 and Thr948) and by the phosphorylation of one inactivating site (Ser899). The latter is targeted by the receptor kinase Feronia, also in response to Rapid Alkalinization Factors (RALFs)^40–42^. Therefore, consistent with the specific role of PLL2 in systemin signalling, the regulatory mechanisms for proton pump inhibition appear to be different for the systemin response and PTI.

## Methods

### Plant material and growth conditions

Tomato (*Solanum lycopersicum* cv. UC82B) and *N. benthamiana* plants were cultivated in growth cabinets at 26 °C, with 16 h photoperiod, 100 µmol m^−2^ s^−1^ light intensity and 75% relative humidity. For seedling growth assays, tomato seeds were sterilized in 2% (*v*/*v*) bleach with 3 drops of Tween 20 per 25 ml and then washed in sterile double-distilled water (ddH_2_O). For germination, seeds were placed on *Arabidopsis thaliana* salts (ATS) medium^37^ containing 1% (*w*/*v*) sucrose. After 1–2 days, seeds that had just germinated were transferred onto either ATS control plates or ATS plates containing 100 nM systemin for another 5–7 days of growth. Root and hypocotyl length were measured using the open source software Fiji (ImageJ v. 2.0.0/1.52p)^43^. The tomato (*S. peruvianum*) cell suspension culture was maintained by weekly transfer to fresh culture medium at 120 rpm and 26 °C (refs. ^18,44^). For the *syr1* mutant cell culture, the medium was supplemented with 75 mg l^−1^ kanamycin.

### Elicitor and wounding treatments

Systemin (AVQSKPPSKRDPPKMQTD), REF1 (ATDRRGRPPSRPKVGSGPPPQNN) and the SlLHA1 C-terminal Thr955 phosphopeptide (GLDIETIQQSYT (ph)V) were obtained from PepMic at >95% purity. Flg22 (QRLSTGSRINSAKDDAAGLQIA) was ordered at >95% purity from GenScript. All peptides were dissolved in ddH_2_O at 1 mM and stored at −20 °C. Chitin (0.5 g) from shrimp shells (Sigma-Aldrich, C9752) was suspended in 10 ml 37% HCl and diluted in 1 l H_2_O. After centrifugation and washing, chitin fragments were suspended in H_2_O to reach 10 mg ml^−1^ (ref. ^44^). For mechanical wounding, the first two true leaves of 3-week-old tomato plants were symmetrically squeezed with a haemostat from both sides of the midrib at each leaflet.

### Phosphoproteomics and data preprocessing

The set-up of phosphoproteomics was adapted from Haj Ahmad et al.^18^; 200 ml cell culture (wild-type and *syr1*) was collected at 0, 1, 2, 5, 15 and 45 min after addition of 10 nM systemin. Samples were collected from six independent batches of cells. Extraction of the microsomal fraction, phosphopeptide enrichment and LC–MS/MS were conducted as previously described^18^. Phosphosites were mapped against *S. lycopersicum* ITAG3.2 by MaxQuant version 2.4.2.0 (ref. ^45^). Overall, 4,804 phosphosites were obtained excluding hits to reverse sequences and potential contaminants and sites with localization probability ≤0.75. Phosphosite intensities in the MaxQuant output table (Phospho (STY)Sites.txt) were normalized and log_2_ transformed in R^46^ version 4.4.0 (all R scripts are available at GitHub (https://github.com/shibalili) under ‘systemin-project’). Data manipulation and visualization were done using tidyverse (v.2.0.0), stringr (v.1.5.1), reshape2 (v.1.4.4), viridis (v.0.6.5), ggpubr (v.0.6.0) and ggplot2 (v.3.5.1) packages^47,48^.

### Exploratory data analysis

The R package limma (v.3.60.4)^49^ was used to analyse differentially phosphorylated phosphosites. For each phosphosite, a linear model was fit to describe its phosphorylation profile over time. Coefficients for each time point were compared to time 0 in multiple pairwise comparison. Sites differentially phosphorylated relative to the same genotype–treatment combination at time 0 were identified at *P* < 0.05. Phosphosites which were differentially phosphorylated (*P* < 0.05) in wild-type cell culture were considered for PCA using the prcomp function in R (v.4.4.0). For analyses that require a complete set of data points for each phosphosite (for example, *k*-means clustering), missing values were imputed using the R package missForest (v.1.5)^50^. A relatively loose cut-off of >10 of maximum 72 data points per phosphosite was applied, which allowed 2,450 (50%) of the phosphosites with variable phosphorylation patterns to be retained for cluster analysis. Gap statistics^51^ was applied to identify the optimal number of clusters as 5 (Extended Data Fig. 2a). Temporal phosphorylation profiles were clustered using the eclust function together with kmeans and euclidean distance from the R package factoextra (v.1.0.7)^52^.

### MapMan enrichment analysis

Functional annotation of proteins was done using MapMan^53^ ontology (BinTree version X4 Release 2.0) downloaded from MapManstore (https://mapman.gabipd.org/mapmanstore), and the *S. lycopersicum* genome annotation ITAG3.2 (ref. ^54^) from PHYTOZOME V13.0 (https://phytozome-next.jgi.doe.gov/). Enrichment analysis was performed with the package hypeR (v.2.2.0)^55^, using hypergeometric enrichment test to determine whether a group of proteins is over-represented.

### Molecular cloning

Polymerase chain reaction (PCR) primers for DNA amplification from plant genomic DNA or plasmid templates are listed in Supplementary Table 3.

To generate expression clones for full-length wild-type *SlPLL2* (*SlPLL2*^*WT*^), a synthetic DNA fragment (Integrated DNA Technology) corresponding to the first 936 bp of PLL2 was inserted into pDONR221 by BP reaction (Invitrogen). The catalytic domain^18^ was added by restriction enzyme cloning. To generate the *SlPLL2*^3A^ and *SlPLL2*^3D^ mutants, a *Cla*I(277)-*Bcl*I(509) fragment of *SlPLL2*^*WT*^ including serines 142, 151 and 160 was replaced with synthetic fragments in which the respective codons had been replaced by GCA for alanine, or GAT for aspartate, respectively. To generate *SlPLL2*^*WT/3A/3D*^*-sfGFP* and *Spot-SlPLL2*^*WT/3A/3D*^ constructs for transient expression in *N. benthamiana*, the *SlPLL2*^*WT/3A/3D*^ variants and the coding sequence of sfGFP were amplified by PCR and inserted into pART7 between the CaMV *35S* promoter and *OCS* terminator. The N-terminal Spot-Tag (PDRVRAVSHWSS) was introduced to *SlPLL2*^*WT/3A/3D*^ by two forward primers, SpotC-PLL5-N (0.5 μM) and SKS-ATG-SpotN (10 μM), by PCR. The two expression cassettes were mobilized by *Not*I digestion and transferred into pART27 (ref. ^56^).

For the BiFC assay, the following fusion constructs were generated: *SlLHA1-YFP*, *SlLHA1-nYFP*, *SlLHA4-YFP*, *SlLHA4-nYFP*, *SlPLL2-cYFP*, *SlSYR1-nYFP* and *SlSERK3B-cYFP*. To this end, the open reading frames (ORFs) of SlLHA1 (Solyc03g113400) and SlLHA4 (Solyc07g017780) and the *SlSYR1* (Solyc03g082470) locus were PCR-amplified from tomato root complementary DNA and genomic DNA, respectively. PCR products were cloned into the *Xho*I and *Cla*I sites of pART7. The ORFs for full-length enhanced yellow fluorescent protein (EYFP, residues 1–240) and the N-terminal fragment of EYFP (nYFP, residues 1–155) were inserted as the *Cla*I- *Xba*I fragment downstream of SlLHA1/4 or SlSYR1 to result in the *SlLHA1/4-YFP*, *SlLHA1/4-nYFP* and *SlSYR1-nYFP* fusions, respectively. Similarly, ORFs of SlPLL2 and SlSERK3B (Solyc01g104970) were fused with the C-terminal fragment of EYFP (cYFP, residues 156–240) to result in *SlPLL2-cYFP* and *SlSERK3B-cYFP*.

For the Co-IP assay, the following expression constructs were generated: *SYR1*, *3xFLAG-LHA1-R* and *3xFLAG-LHA4-R*, in addition to *SlPLL2*^*WT*^*-sfGFP* described above. A genomic fragment comprising *SYR1* (Solyc03g082470) was PCR-amplified from genomic DNA and cloned into the multiple cloning site between the 35S promoter and the 19S terminator in the binary vector pMB35S (ref. ^57^) using the *Eco*RV and *Xba*I sites. The regulatory domain of SlLHA1 (aa 864–956) and SlLHA4 (aa 859–952) were PCR-amplified from full-length cDNA templates. The 3xFLAG tag sequence was included in the PCR primers and added at the N-terminus of SlLHA1 and SlLHA4.

The yeast complementation assay required expression vectors for *SlLHA4* and *SlLHA1* under control of the endogenous *PMA1* promoter. For this purpose, the *AtAHA2* ORF in the previously described *pPMA1:AtAHA2* expression plasmid pMP1745 was replaced by *SlLHA4* or *SlLHA1* by *Xho*I-*Nde*I digestion^30^. *SlPLL2* and its phospho-variants were cloned into the pMP1612 expression vector using *Not*I^30^.

For CRISPR/Cas9-mediated genome editing, single guide RNA (sgRNA) constructs were generated in the pKSE401 binary vector comprising the *pCaMV35S::Cas9* expression construct^58^. CRISPRdirect (https://crispr.dbcls.jp) was used to select guide RNAs that included a restriction site at the location of the double strand break to facilitate subsequent genotyping. sgRNA expression cassettes were generated by PCR using primers containing the sgRNA sequence and a *Bsa*I site for golden gate cloning, and pCBCT1T2 and/or pCBCT2T3 as the template. Primer sequences are listed in Supplementary Table 4. The resulting *pKSE401-gSYR1* and *pKSE401-gPLL2* editing constructs contained two and three sgRNAs for the genomic *SlSYR1* and *SlPLL2* loci, respectively. All constructs were verified by sequence analysis using SnapGene (v. 7.0.2).

### CRISPR/Cas9 mutagenesis

Cotyledons of 10-day-old etiolated tomato (*S. lycopersicum* cv. UC82b) seedlings were used as explants for *Agrobacterium tumefaciens* (GV3101)-mediated transformation. They were wounded several times across the mid rib, placed upside down on conditioning medium (Murashige and Skoog Basal Salts with minimal organics (MSMO), 1 mg l^−1^ naphthaleneacetic acid, 0.1 mg l^−1^ 6-benzylaminopurine, 6% (*w*/*v*) agar) and incubated for 2 to 3 days at 22 °C in the dark. Agrobacteria carrying the CRISPR/Cas9 gene editing constructs were grown in 10 ml yeast extract beef broth with appropriate antibiotics and 0.2 mM acetosyringone at 28 °C, 220 rpm. In the exponential growth phase, cells were collected by centrifugation and resuspended in 10 mM MgSO_4_ to result in optical density at 590 nm (OD_590_) = 0.5–1.0. Cotyledon explants were covered with a drop of the bacterial suspension and incubated for 2 days at 22 °C in darkness. Explants were then rinsed in MSMO, blotted dry, placed right-side up onto selection medium (MSMO, 9.6 mg l^−1^ thiamine, 1 mg l^−1^ nicotinic acid, 1 mg l^−1^ pyridoxine, 1 mg l^−1^ *trans*-zeatin, 250 mg l^−1^ timentin, 35 mg l^−1^ kanamycin, 0.6% (*w*/*v*) agar) and incubated at 22–25 °C, 16 h photoperiod. Explants were transferred to fresh plates every week, increasing kanamycin concentration stepwise from 35 to 50 and then 100 mg l^−1^. Shoots were transferred to rooting medium (same as selection medium with 0.1 mg l^−1^ indole-3-acetic acid replacing *trans*-zeatin, 20 mg l^−1^ kanamycin, 500 mg l^−1^ vancomycin) when they were 2–3 cm tall. Regenerated plantlets were transferred to soil, maintained at high humidity for a couple of days and transferred to the green house.

The *syr1* mutant cell culture was also generated by CRISPR/Cas9 genome editing. The editing construct was linearized with *Pme*I and shot into *S. peruvianum* cells using the PDS1000/He Biolistic Particle Delivery System (BioRad)^59^. Transformed cells were selected on 75 mg ml^−1^ kanamycin for callus growth. Suspension cultures were established from calli that were confirmed by PCR to carry the editing construct and genotyped as follows.

### Genotyping of CRISPR/Cas9 mutants

Genomic DNA was isolated from wild-type tomato plants or *S. peruvianum* calli and corresponding CRISPR/Cas9 transformants. For transgenic plants, three independent *syr1* and *slpll2* mutants were identified by PCR and sequenced in the T0 generation (primers for genotyping are listed in Supplementary Table 4). Homozygous mutants from the segregating Cas9-free T2 progeny were used for experiments (Supplementary Table 1). For transgenic cell cultures, genotyping was done by cloning the PCR-amplified target loci in pCR2.1-TOPO (Thermo Fisher Scientific). Eight clones per cell culture were sequenced revealing different mutations at the target site of the sgRNA and confirming the absence of the wild-type sequence (Supplementary Table 1).

### RNA isolation and reverse transcriptase quantitative PCR

Total RNA was extracted from leaves of 3-week-old tomato plants with TRIzol reagent (Mobitec, FP312) according to the manufacturer’s instructions. One microgram RNA digested with DNaseI (Thermo Scientific, ENO521) was reversely transcribed using RevertAid reverse transcriptase (Thermo Fisher Scientific, EPO442) and oligo(dT) primers. Real-time quantitative PCR (qPCR) was performed in a Bio-Rad CFX Connect real-time PCR instrument (Bio-Rad) with SYBR-Green (Invitrogen, S7563). CFX Manager (BioRad) was used for data acquisition and analysis. Reverse transcriptase qPCR (RT-qPCR) primers are listed in Supplementary Table 5. Target gene expression was analysed by delta–delta Ct method and normalized to the expression level of three housekeeping genes, *UBQ10*, *ACTIN2* and *EF1α*.

### Transient protein expression in *N. benthamiana*

For transient expression experiments, cultured agrobacteria carrying binary vectors with expression constructs for the protein(s) of interest and the P19 silencing suppressor were mixed to result in optical density at 600 nm (OD_600_) = 0.5. When PLL2 mutants were expressed for activity assays, the OD_600_ ratio (PLL2^WT/3A/3D^ or free GFP: P19) was 9:1. *N. benthamiana* lacks a functional systemin perception system. Therefore, to reconstitute systemin signalling in *N. benthamiana*, a SYR1-eGFP expression construct kindly provided by G. Felix (University of Tübingen) was always co-infiltrated with the PLL2 constructs (PLL2^WT/3A/3D^/SYR1/P19 = 8:1:1). For Co-IP, the OD_600_ ratio was PLL2^WT/3A/3D^/LHA1/4/P19 = 4.5:4.5:1 or PLL2^WT^/LHA1/SYR1/P19 = 4:4:1:1. The infiltration buffer contained 10 mM MgCl_2_, 10 mM 2-(*N*-morpholino)ethanesulfonic acid, pH 5.6 and 150 μM acetosyringone. Leaves were collected 3 days after infiltration.

### Alkalinization assay

Medium alkalinization in response to systemin (10 nM), flg22 (20 nM) and ddH_2_O as the control was analysed in *S. peruvianum* wild-type and *syr1* cell suspensions 7 days after subculture using a pH meter in 10 ml aliquots^44^.

### ROS burst

ROS production in response to systemin (30 nM), flg22 (30 nM), REF1 (30 nM), chitin (50 µg ml^−1^) and ddH_2_O as the control was analysed in 4 mm leaf discs of 4-week-old tomato or *N. benthamiana* plants using a luminescence-based assay^60^.

### MAPK activation

Three-week-old tomato plants with two true leaves were dipped briefly into 1 μM systemin or REF1 in 0.05% (*v*/*v*) silwet-77 or the solvent alone. At the indicated time points, 100 mg tissue samples were ground in 100 μl extraction buffer (100 mM NaCl, 50 mM Tris–HCl, pH 7.5, 0.5% (*v*/*v*) Triton X-100, 10 mM β-mercaptoethanol, 1:1,000 Protease Inhibitor Mix P (SERVA, 39103.02), 1:2,000 Phosphatase Inhibitor Cocktail 2 and 1:2,000 Phosphatase Inhibitor Cocktail 3 (Sigma, P5726 and P0044)) using a bead beater (TissueLyzer LT; QIAGEN). The extract was cleared by centrifugation. Protein samples were separated by 10% (*w*/*v*) SDS–PAGE (sodium dodecyl sulfate–polyacrylamide gel electrophoresis) and blotted to nitrocellulose. Blots were developed using anti-pERK1/2 (1:5,000; Cell Signaling) and goat anti-rabbit IgG (1:10,000; Millipore) antibodies by enhanced chemiluminescence detection in a LICORbio Odyssey XF imager with Image Studio (version 5.5) software.

### In vitro phosphatase assay

The pART27-based expression constructs harbouring phosphatase (SlPLL2^WT^, SlPLL2^3A^ and SlPLL2^3D^)-sfGFP were agro-infiltrated into *N. benthamiana* leaves. Three days after infiltration, 100 mg leaf samples were collected and ground in 100 μl extraction buffer (100 mM NaCl, 50 mM Tris–HCl, pH 7.5, 0.5% (*v*/*v*) Triton X-100, 10 mM β-mercaptoethanol, 1:1,000 Protease Inhibitor Mix P) using a bead beater (TissueLyser LT; Qiagen). The extracts were cleared by centrifugation. An aliquot of the supernatant was used for the quantification of phosphatase expression levels by anti-GFP western blot analysis. The remainder was subjected to GFP-trap (Chromotek, gtma20) to immuno-precipitate SlPLL2 variants according to the manufacturer’s instructions. On-bead phosphatase activity assays were performed using the Serine/Threonine Phosphatase Assay Kit (Promega, V2460), with the bead volume adjusted to result in equal amounts of enzyme for each of the PLL2 variants (Extended Data Fig. 3). The phospho-peptide GLDIETIQQSYT(ph)V, custom-synthesized at >95% purity (PepMic), was used as the substrate at 100 µM.

### Co-IP

Constructs bearing phosphatase-sfGFP fusion constructs and the 3xFLAG-tagged regulatory domains of LHA1 and LHA4 were transformed in *A. tumefaciens* and co-infiltrated in *N. benthamiana* leaves in the combinations indicated. For elicitor treatment, leaves were infiltrated with 100 nM systemin or 100 nM flg22, collected and flash-frozen in liquid nitrogen within 2 min. Two infiltrated leaves (~2 g) were ground in 2 ml cold IP buffer (150 mM NaCl, 50 mM Tris–HCl, pH 7.5, 10% (*v*/*v*) glycerol, 5 μM DTT, 0.5% (*v*/*v*) IGEPAL detergent, 1 mM PMSF, 1:1,000 Protease Inhibitor Mix P from SERVA) on ice. The extract was cleared by centrifugation (16,100*g*, 15 min, 4 °C), and 50 μl of the supernatant was collected as input control. The remaining extract was incubated with 25 µl anti-FLAG M2 affinity gel (Sigma-Aldrich, A2220) at 4 °C for 3 h. After 5 washing steps in 150 mM NaCl, 50 mM Tris–HCl, pH 7.5 and 10% (*v*/*v*) glycerol, the immunoprecipitated proteins and input controls were subjected to SDS–PAGE followed by western blot. Blots were developed with anti-Flag-HRP (1:5,000, Sigma-Aldrich, A8592) and anti-GFP (1:10,000, Invitrogen) antibodies.

### Yeast complementation assay

Expression constructs for *SlLHA1* or *SlLHA4* under control of the yeast *PMA1* promotor in pMP1745, and the *SlPLL2* variants in pMP1612 were co-transformed into *Saccharomyces cerevisiae* strain RS-72 (*MATα*; *ade11-100*, *his4-519*, *Leu2-3*, *112*, *pPMA1:GAL1*) using the lithium-acetate method^30^. The empty vectors were used as negative controls. Transformed cells were grown on galactose medium (SG + His, pH 5.5). Galactose induces the expression of the yeast proton pump PMA1 from the *GAL1* promoter. After transfer to glucose medium (SD + His, pH 5.5), growth depends on the activity of the plasmid-borne tomato proton pumps LHA1 and LHA4. To assess the effect of the PLL2 variants on tomato proton pump activity, three single yeast colonies from one transformation were diluted in liquid glucose medium to OD_600_ = 0.1 and 0.01. Five microlitres of each dilution were spotted on selective media. Plates were incubated at 30 °C and imaged after 2 days (galactose plates) or 3 days (glucose plates), respectively. The experiment was repeated three times with cells from independent transformations.

### Isolation of the yeast microsomal fraction

A single colony was grown in 10 ml liquid galactose medium (SG + His, pH 5.5) and incubated overnight at 200 rpm, 30 °C. The next day, it was transferred to 250 ml galactose medium and incubated overnight as before. The third day, this yeast culture was pelleted at room temperature, 800*g* for 10 min, then inoculated into 500 ml liquid glucose medium (SD + His, pH 5.5) for 20 h at 200 rpm, 30 °C. Finally, cells were pelleted at 5,000*g* and resuspended in 6 ml cold water. Yeast cells (6 ml) were then lysed in 3 ml homogenization buffer (1 vol 0.5 M Tris, pH 7.5, 1/100 vol 0.5 M EDTA, 1/500 vol 0.5 M DTT), 30 µl PMSF and 30 µl pepstatin with 23 g glass beads by vortexing. Extracts were cleared by centrifugation (15 min, 10,000*g*, 4 °C). Microsomal membranes were collected from the supernatant by ultracentrifugation at 50,000*g*, 4 °C for 45 min. STED10 (10% (*w*/*v*) sucrose, 0.1 M Tris–HCl, pH 7.5, 1 mM EDTA, 1 mM DTT) was used to fill and balance the tubes. Membranes were resuspended in GTED20 (23% (*v*/*v*) glycerol, 0.1 M Tris–HCl, pH 7.5, 1 mM EDTA, 1 mM DTT). Protein concentration was determined using the Bradford method with γ-globulin as the reference.

### Western blot overlay (far-western blot)

Microsomal fractions (100 µg) were denatured in SDS–PAGE sample buffer at 50 °C for 10 min and separated by 8% (*v*/*v*) SDS–PAGE (40% (*w*/*v*) acrylamide 37:1, 1.5 M Tris–HCl, pH 8.85, 10% SDS, 4% (*v*/*v*) TEMED, 12% (*v*/*v*) APS) and blotted to nitrocellulose membranes. For the detection of LHA1 and LHA4, membranes were blocked with 5% (*w*/*v*) skim milk in wash buffer (25 mM Tris, 150 mM NaCl and 0.1% (*v*/*v*) Tween 20, pH 7.4). A polyclonal serum against AtAHA2 C-terminus (aa 851–949; 1:5,000) and anti-rabbit IgG-HRP (1:10,000; Millipore) were used as the first and secondary antibodies, respectively. To assess the phosphorylation status of the penultimate threonine at the C-terminus of the proton pumps, membranes were blocked with 5% (*w*/*v*) skim milk in far-western buffer (20 mM MES, 130 mM NaCl, 10 mM MgSO_4_ and 100 µM CaCl_2_, pH 6.5) for 1 h at room temperature. Then membranes were transferred into 10 ml far-western buffer containing 40 μg RGS-His epitope tagged 14–3–3 (GF14ø) protein^28^ and 5 µM fusicoccin (10 mM stock in 96% ethanol, Santa Cruz Biotechnology, sc-200754) for 1 h at room temperature. Then 14–3–3 protein binding was detected using a monoclonal anti-RGS-6x-His antibody (1:1,000; Qiagen) followed by anti-mouse-HRP (1:10,000; Millipore) as the primary and secondary antibodies, respectively.

### Insect feeding assays

*M. sexta* eggs were collected and kept in a climate chamber at 16 h/26 °C and 8 h/24 °C light–dark cycle until the larvae hatched. Two freshly hatched neonates were placed on the second true leaf (from bottom to top) of 4-week-old tomato plants and enclosed in white organza bags. The developing larvae were moved onto younger leaves when the tissue was consumed. Larval mass was measured every 2 days until all leaves were consumed.

### Quantification of proteinase inhibitor (PI-II) activity

Proteinase inhibitor activity was assayed in tomato leaf extracts as soybean trypsin inhibitor (STI) equivalents using a radial diffusion assay^61^. Briefly, leaf disc samples (150 mg) were collected into 2 ml screw-cap tubes containing 0.6 g ZrO_2_ beads (2.8–3.2 mm; Mühlmeier, Germany) and flash-frozen in liquid nitrogen. Samples were homogenized in 500 µl extraction buffer (50 g l^−1^ PVPP, 18.6 g l^−1^ Na_2_EDTA, 5 g l^−1^ Na-diethyldithiocarbamate-trihydrate, 2 g l^−1^ phenylthiourea in 0.1 M Tris–HCl, pH 7.6 for 150 µg of tissue) in a Fisherbrand Bead Mill 24 homogenizer at 4 m s^−1^ for 2 × 30 s. The extract was cleared by centrifugation (15 min, 16,100*g*, 4 °C). The supernatant was filled into ø 0.4 mm wells punched into 12 × 12 cm plates containing 25 ml of 1.8% (*w*/*v*) agar with 2 mg bovine trypsin (Sigma-Aldrich, 3708985001) in 0.1 M Tris–HCl, pH 7.6). After 18 h at 4 °C, 25 ml substrate solution (6 mg *N*-acetyl-dl-phenylalanine-naphthyl ester, 12 mg Fast Blue B salt in 0.1 M Tris/20% (*v*/*v*) dimethyl formamide) was added to stain for trypsin activity during 1 h at 37 °C. The diameter of the clear inhibition zones surrounding each well was used to calculate trypsin protease inhibitor activity using a standard curve with STI (Sigma-Aldrich, T9128) as the reference. Sixteen plants per genotype were analysed in two technical replicates.

### Rhizosphere acidification and alkalinization assays

Five- to six-day-old wild-type and *pll2* tomato seedlings grown on ATS plates were transferred onto pH indicator plates containing 0.002% (*w*/*v*) bromocresol purple (BCP) in water–agar (0.5% (*w*/*v*) agar, pH 6.5 adjusted with KOH). Pictures were taken after 24 and 48 h of incubation. To monitor systemin-induced alkalinization of the rhizosphere, seedlings were transferred from ATS plates to indicator plates adjusted to pH 5.5 with acetic acid. They were then sprayed with 1 μM systemin or water as the control. To analyse the effect of fusicoccin on systemin-induced alkalinization of the rhizosphere, seedlings were sprayed with 10 µM fusicoccin or ddH_2_O as the control and incubated on ATS plates for 15 min before they were transferred to pH 5.5 indicator plates for systemin treatment as above. Pictures were taken before and 30 min after systemin application.

### Phylogenetic analysis

Phylogenetic trees of *S. lycopersicum* and *Arabidopsis thaliana* proteins in the P-type H^+^-ATPase and POLTERGEIST-LIKE families were generated using PhyloGenes v4.1(www.phylogenes.org)^62^. SlLHA1 (Solyc03g113400), SlLHA4 (Solyc07g017780) and SlPLL2 (Solyc06g076100) are highlighted in red in Extended Data Figs. 4b (SlLHA1 and 4) and 2c (PLL2), respectively.

### Confocal microscopy and image acquisition

Subcellular localization of GFP-fusion proteins was imaged on a Zeiss LSM980, and BiFC images were acquired on a Zeiss LSM700 confocal microscope. GFP and FM 4–64 fluorescence were detected using an LD LCI Plan-Apochromat ×40/1.2 lmm Korr DIC M27 objective with a 488 nM argon laser. YFP fluorescence was detected using a Plan-Apochromat ×20/0.8 objective with a 514 nM excitation line. Representative confocal images were acquired as Z-stacks and rendered as three-dimensional projections using a series of optical sections with Zen software (blue edition (v3.6) and black edition (2020) for the LSM980 and LSM700, respectively) or open source software Fiji (ImageJ version 2.0.0/1.52p)^43^. Insect images were taken with a Nikon D5600 DSLR camera with 18–55 mm f/3.5–5.6 auto focus-P Nikkor Zoom lens.

### Statistical analysis

Statistical tests in phosphoproteomics were performed using R packages (Limma, factoextra, hypeR). Details of other statistical analyses are indicated in the figure legends. Unpaired two-tailed Student’s *t*-test and one-way analysis of variance (ANOVA) with Tukey’s multiple comparisons test were performed in GraphPad Prism 9.0 (GraphPad Software).

### Reporting summary

Further information on research design is available in the Nature Portfolio Reporting Summary linked to this article.

## Supplementary information

Supplementary Information Supplementary Tables 1 and 3–5, Figs. 1–4 and note.

Reporting Summary

Supplementary Table 2 List of identified phospho-peptides along with the corresponding gene ID, protein annotation and *k*-means cluster.

## Source data

Source Data Fig. 1 Statistical source data.

Source Data Fig. 2 Unprocessed western blots and/or gels with replicates.

Source Data Fig. 2 Statistical source data.

Source Data Fig. 3 Unprocessed western blots and/or gels with replicates.

Source Data Fig. 3 Statistical source data.

Source Data Extended Data Fig. 3 Unprocessed western blots and/or gels with replicates.

Source Data Extended Data Fig. 7 Statistical source data.

Source Data Extended Data Fig. 8 Unprocessed western blots and/or gels with replicates.

Source Data Extended Data Fig. 8 Statistical source data.

## Data Availability

*S. lycopersicum* genome annotation ITAG3.2 from PHYTOZOME V13.0 (https://phytozome-next.jgi.doe.gov/) was used for phospho-site mapping. MapMan ontology from MapManstore (https://mapman.gabipd.org/mapmanstore) was used for functional annotation and over-representation analysis. The mass spectrometry proteomics data have been deposited to the ProteomeXchange Consortium via the PRIDE^63^ partner repository with dataset identifiers PXD054229 and PXD062081. Raw data are included with the paper as source data and are available via figshare at 10.6084/m9.figshare.28175321 (ref. ^64^). Source data are provided with this paper.

## Code availability

R scripts for phosphoproteomic analysis and the linear regression plot of Fig. 2a are available via GitHub at https://github.com/shibalili/systemin-project. All bioinformatic tools used in this study are cited in Methods.
