# Supplementary material for: Poltergeist-Like 2 (PLL2)-dependent activation of herbivore defence distinguishes systemin from other immune signalling pathways
Source: Nat Plants. 2025 Jul 4;11(7):1270–81. doi: 10.1038/s41477-025-02040-7 (PMC12283378; doi:10.1038/s41477-025-02040-7)
Supplement: Supplementary file 1 — Supplementary Tables 1 and 3–5, Figs. 1–4 and note. [file 41477_2025_2040_MOESM1_ESM.pdf]

nature plants

Supplementary information https://doi.org/10.1038/s41477-025-02040-7

Poltergeist-Like 2 (PLL2)-dependent activation of herbivore defence distinguishes systemin from other immune signalling pathways

In the format provided by the authors and unedited

Supplementary Note: Missing values in label-free quantitative proteomics

A large number of phospho-sites showed a transient drop in abundance at 1 and 2 min after systemin treatment (Fig. 1d). This also included the peptides phosphorylated at serines 142, 151 and Ser160 of PLL2. Two of these sites (Ser142 and Ser160) were undetectable at the 1 min time point (Fig. 1f).

Technical issues with respect to peptide recovery and/or quantification cannot explain the apparent drop in intensity or ‚disappearance' of peptides at the 1 min time point, because the average number of phospho-peptides detected in the six biological replicates was the same for all time points, and the average intensity of these peptides was also the same for at least 5 of the 6 replicates.

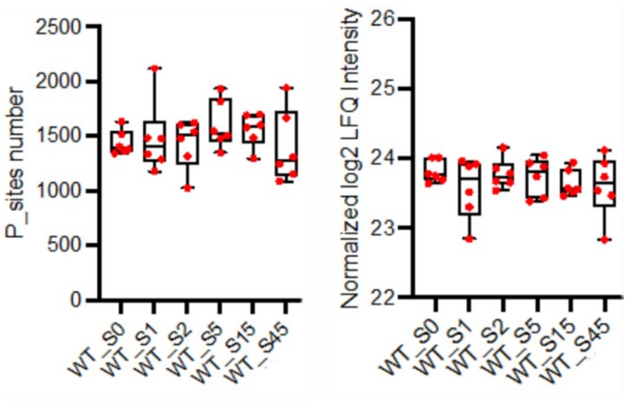

The drop in intensity is thus explained by systemin-induced transient dephosphorylation. It was highly significant for Ser151 of PLL2 (Fig. 1f). The data are not as clear for Ser142 and Ser160, because there are no measurements at 1 min after treatment, and several values are missing also at other time points (Fig. 1f).

Missing values (MVs), typically between 20 % and 50% of the total possible peptide values, are a general problem in label-free quantitative proteomics[1]. There is a large body of literature on the MV problem and how to deal with it. Generally, two types of missingness are distinguished: First, data can be missing at random (MAR), which is mostly due to technical issues, such as poor-digestion resulting in mis-cleaved peptides, poor ionization efficiency, the semi-stochastic way in which ions are chosen for fragmentation, or bad peptide-spectrum-matches[2]. In this scenario, MVs will be randomly distributed over the time course and the different replicates. Second, data can be missing not at random (MNAR) and this is mostly due to biological reasons. Peptides may not be present at all, or at levels below the detection limit of the instrument used. In this case, peptide intensities are left-censored and missingness depends on peptide abundance[2-4]. Hence, if phospho-peptide abundance changes over time, MVs will not be randomly distributed over the time course but rather be concentrated at specific time point(s). This will be most prevalent for peptides that are detected with low intensities (e.g. due to poor ionization properties), because the intensity may drop below the detection limit when peptide abundance decreases. This is what we observed for the Ser142 and Ser160 phospho-sites of PLL2.

The figure below shows the total number of phospho-peptides detected at the t=1 min time point after systemin treatment in different windows of log2 ion intensity (18-20, 20-22, 22-24, 24-26, ….), and in how many of the six biological replicates (1/6, 2/6, 3/6 …) they occurred. It becomes apparent that for intensities > 24, the majority of peptides (194) can be detected in all 6 biological replicates, but such high-intensity peptides are not very frequent. In contrast, for peptides <22 the majority (366) is found in only 1/6 replicates, and peptides <20 are essentially undetectable. The sharp drop in

detectability at the lower end of the intensity range indicates that the data are left-censored and that MNAR contributes to missingness in our data.

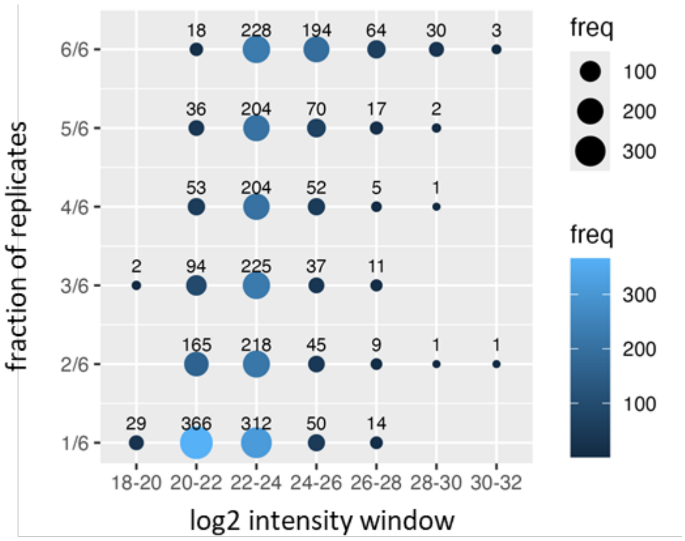

The two peptides containing the Ser142 and Ser160 phospho-sites had a normalized ion intensity of about 23 and 22 at t=0 min, respectively. Therefore, they cannot be expected to be found in all biological replicates. If such low intensity peptides are dephosphorylated resulting in an intensity <20, they are essentially undetectable, explaining absence of these peptides at t=1 min after systemin treatment.

We conclude that PLL2 was transiently dephosphorylated in response to systemin treatment at Ser151, and likely also at serines 142 and Ser160. The latter two sites were undetectable at 1 min after systemin treatment, consistent with a decrease in abundance due to dephosphorylation. However, since these phospho-peptides were not reliably detected in all biological replicates, with some data points missing also at other time points, the evidence for dephosphorylation is not as strong as for the Ser151 phospho-site.

References:

1 Webb-Robertson, B.-J. M. *et al.* Review, Evaluation, and Discussion of the Challenges of Missing Value Imputation for Mass Spectrometry-Based Label-Free Global Proteomics. *Journal of Proteome Research* **14**, 1993-2001 (2015).

2 Jin, L. *et al.* A comparative study of evaluating missing value imputation methods in label-free proteomics. *Scientific Reports* **11**, 1760 (2021).

3 Karpievitch, Y. V., Dabney, A. R. & Smith, R. D. Normalization and missing value imputation for label-free LC-MS analysis. *BMC Bioinformatics* **13**, S5 (2012).

4 Karpievitch, Y. *et al.* A statistical framework for protein quantitation in bottom-up MS-based proteomics. *Bioinformatics* **25**, 2028-2034 (2009).

Table S1 - Mutations identified in the *S. peruvianum syr1* cell culture, and in *slpll2* tomato plants

CRISPR/Cas9 construct	Mutation at 1st gRNA	Mutation at $2^{nd}/3^{rd}$ gRNA	Mutation at protein level
Solyc03g082470 (SYR1)<br>1115 aa	+1 bp	-8 bp	truncated protein of 100 aa
	-1 bp	-8 bp	truncated protein of 106 aa
	-3 bp	-1 bp	truncated protein of 427 aa
	-7 bp	-19 bp	truncated protein of 104 aa
	-7 bp	-54 bp	truncated protein of 104 aa
	-6 bp	-2 bp	truncated protein of 426 aa
	-964 bp		truncated protein of 96 aa
Solyc06g076100 (SlPLL2)<br>709 aa	-1 bp	2nd gRNA: -2 bp<br>3rd gRNA: WT	truncated protein of 219 aa
	-595 bp		truncated protein of 147 aa
	+1 bp	2nd gRNA: +1 bp<br>3rd gRNA: WT	truncated protein of 145 aa

**Table S3 - PCR cloning primers**			
Primer name	Gene ID	Sequence (5'-3')	Description
76100NcoI241F	Solyc06g076100	ATACCATGGCACATCACCATCACCATCACTTCAGCAGTGAGTGTAGTTTG	PLL2 catalytic domain
76100XhoI708R	Solyc06g076100	AATTCTCGAGTTATGCACTGGATCTCCATATTC	PLL2 catalytic domain
SKpn+ATG-PLL5	Solyc06g076100	cccgggtaccATGGGAAACGGTGTAGGAAAaCTTAGTGTTTG	For tobacco transient expression:PLL2-sfGFP
PLL5Cterm-*-BamR	Solyc06g076100	cccgggatccTGCACTGGATCTCCATATTCTTCC	For tobacco transient expression:PLL2-sfGFP
SKS-ATG-SpotN	Solyc06g076100	cccgggtaccGTCGACATGCCTGATAGAGTGAGGGCTGTTTCTCATTGGA	For tobacco transient expression:Spot-PLL2
SpotC-PLL5-N	Solyc06g076100	GAGGGCTGTTTCTCATTGGAGTTCAGGAAACGGTGTAGGAAAaCTTAGTG	For tobacco transient expression:Spot-PLL2
PLL5-591R	Solyc06g076100	AGCTCTTTGTAGAACCCGAAAG	For tobacco transient expression:Spot-PLL2
AHASl07g017780_XhoI_ATG_F	Solyc07g017780	ccccCTCGAGATGGCGAAAGCTATAAGCCT	For BiFc: 5'LHA4 (with NcoI-cut 3'LHA4 for full length)
AHA Solyc07g017780-1315F	Solyc07g017780	AAGGAAGATGTCAGGAGAAAGGTTC	For BiFc: 5'LHA4 (with NcoI-cut 3'LHA4 for full length)
AHA Solyc07g017780-1616R	Solyc07g017780	GATGCTGATGGGTACATGTTTGTTC	For BiFc: 3'LHA4 (with NcoI-cut 5'LHA4 for full length)
AHASl7g017780ClaIno*2856R	Solyc07g017780	ccccATCGATAACTGTATAATGCTGCTGGATCGTTT	For BiFc: 3'LHA4 (with NcoI-cut 5'LHA4 for full length)
LHA1-Sl03g113400_Sal1-ATG	Solyc03g113400	ccccGTCGACGTCATGGCGGAAAAGCCTGAAGTT	For BiFc: 5'LHA1 (with NcoI-cut 3'LHA1 for full length)
Solyc03g113400-1507R	Solyc03g113400	CTCCAAGGTTTAAAGCCCTCCT	For BiFc: 5'LHA1 (with NcoI-cut 3'LHA1 for full length)
Solyc03g113400-1299F	Solyc03g113400	CAAGTCTGACATAGAGCGTAGAGT	For BiFc: 3'LHA1 (with NcoI-cut 5'LHA1 for full length)
LHA1-3g113400ClaIno*3324R	Solyc03g113400	ccccATCGATAACGGTGTATGACTGCTGAATTGT	For BiFc: 3'LHA1 (with NcoI-cut 5'LHA1 for full length)
spotPLL5_XhoKpnI_ATG_F	Solyc06g076100	CCGCTCGAGgtaccATGCCTGATAGAGTGAGGGCT	For BiFc
spotPLL5_Cla_no*-2160R	Solyc06g076100	CCCCATCGATTGCACTGGATCTCCATATTCTTCCC	For BiFc
SalI_gSYR1_Fw	solyc03g082470	ccgtcgacatgttcttgtttgatgttgt	clone into pART7-nYFP for BiFC
gSYR1_ClaI_Rv	solyc03g082470	gtctatcgattaactcgacaagagaccttacta	clone into pART7-nYFP for BiFC
XhoI_SERK3B_Fw	Solyc01g104970	cgatctcgagatgatggatcaatgggt	clone into pART7-cYFP for BiFC
SERK3B_ClaI_Rv	Solyc01g104970	gtctatcgattcttggccctgataactcat	clone into pART7-cYFP for BiFC
SYR1_EcoRV_Fw	Solyc03g082470	GCTGATATCATGTTCTTGTTTGATGTTGTTCA	For CoIP
SYR1_XbaI_Rv	Solyc03g082470	CAATCTAGATTATAACTCGACAAGAGACCTTACT	For CoIP
FLAG-ATG_XhoI	3xFLAG	ccggCTCGAGATGGACTACAAGGACGATGATGATAAGGATTATAA	For CoIP
FLAG-junction	3xFLAG	GGACGATGATGATAAGGATTATAAAGATGATGACGACAAGGACTACAAGG	For CoIP
1.5Flag_AHA-2733F	3xFLAG;Solyc07g017780	ACGACAAGGACTACAAGGACGACGATGACAAAAACAAGACTGCTTTCACC	For CoIP
AHA_Hind-2856R	Solyc07g017780	ccggAAGCTTTTAAACTGTATAATGCTGCTGGATC	For CoIP
1.5Flag_LHA1-2819	3xFLAG;Solyc03g113400	ACGACAAGGACTACAAGGACGACGATGACAAACAAAGGATTGCTTTCACC	For CoIP
LHA1_Hind-3324R	Solyc03g113400	ccggAAGCTTTTAAACGGTGTATGACTGCTGAATT	For CoIP
AHA Sl07g017780_Xho-Kozak.1	Solyc07g017780	TCACTCTCGAGtaccaATGGCcAAAGCTATAAGCCTCGAAGAG	For yeast complementation
AHA Sl07g017780*_NdeI.2869R	Solyc07g017780	TGACACATATGCTAAACTGTATAATGCTGCTGGATCGTT	For yeast complementation
LHA1-Sl03g113400_Sal1-Kozak.1	Solyc03g113400	TCACTGTCGACtaccaATGGCcGAAAAGCCTGAAGTTCTGGATGC	For yeast complementation
LHA1-Sl03g113400*_NdeI.3144R	Solyc03g113400	TGACTCATATGTTAAACGGTGTATGACTGCTGAATTGT	For yeast complementation
PLL5_NotI-Kozak.1	Solyc06g076100	TCACTGCGGCCGCtaccaATGGGcAACGGTGTAGGAAAaCTTAGTGTTTG	For yeast complementation
PLL5*_NotI.2127R	Solyc06g076100	TGACTGCGGCCGCTTATGCACTGGATCTCCATATTCTTCC	For yeast complementation

Table S4 - Primers used for genome editing and genotyping				
restriction site highligted in red; targeting sequence of sgRNA in bold				
Primer name	Target Gene ID	Sequence (5'-3')	Restriction enzyme	Description
DT824701-BsF	Solyc03g082470	ATATATGGTCTCGATTg**CGGTAACTTGCCGGTCAAT**GTT	*Hpa* II	CRISPR:pKSE401-gSYR1 :1st gRNA
DT824701-F0	Solyc03g082470	Tg**CGGTAACTTGCCGGTCAAT**GTTTTAGAGCTAGAAATAGC	*Hpa* II	CRISPR:pKSE401-gSYR1 :1st gRNA
DT824702-R0	Solyc03g082470	AAC**CGTTTGGTCTCCCTTGATC**cAATCTCTTAGTCGACTCTAC	*Bsa* I	CRISPR:pKSE401-gSYR1 :2nd gRNA
DT824702-BsR	Solyc03g082470	ATTATTGGTCTCGAAAC**CGTTTGGTCTCCCTTGATC**cCAA	*Bsa* I	CRISPR:pKSE401-gSYR1 :2nd gRNA
U6-26p-F		TGTCCCAGGATTAGAATGATTAGGC		2 sgRNA CRISPR:pKSE401 construct: 2gRNA against SYR1
U6-29p-R		AGCCCTCTTCTTTCGATCCATCAAC		2 sgRNA CRISPR:pKSE401 construct: 2gRNA against SYR1
82470CRISPRF1-172	Solyc03g082470	AAGGGTGTTACCTGCTATTCAGAC		Genotyping:*syr1* ;1st gRNA
82470CRISPRR1-456	Solyc03g082470	GTAACCCAACTCAAGGTATACAAGC		Genotyping:*syr1* ;1st gRNA
82470CRISPRF2-1071	Solyc03g082470	CACTTCTCTTGCTGAGATAAGTCTCG		Genotyping:*syr1* ;2nd gRNA
82470CRISPRR2-1427	Solyc03g082470	AGTTTAGAAGGAATCTGTCCACTGAAG		Genotyping:*syr1* ;2nd gRNA
403-Sl06g076100DT1-BsF	Solyc06g076100	ATATATGGTCTCGATTg**GTTCCGGCTTCGGGCCCTA**GTT	ApaI	CRISPR:pKSE401-gPLL2:1st gRNA
403-Sl06g076100DT1-F0	Solyc06g076100	Tg**GTTCCGGCTTCGGGCCCTA**GTTTTAGAGCTAGAAATAGC	ApaI	CRISPR:pKSE401-gPLL2:1st gRNA
997-Sl06g076100DT2-BsF	Solyc06g076100	ATATTATTGGTCTCAAGATTg**CCATCTCCGAGTTTGGCCG**GTT	EaeI	CRISPR:pKSE401-gPLL2:2nd gRNA
997-Sl06g076100DT2-F0	Solyc06g076100	Tg**CCATCTCCGAGTTTGGCCG**GTTTTAGAGCTAGAAATAGC	EaeI	CRISPR:pKSE401-gPLL2:2nd gRNA
1250-Sl06g076100DT3-R0	Solyc06g076100	AAC**TTCAGCTGTTCCTTCAATC**cAATCACTACTTCGTCTCTAACCAT	PvuII	CRISPR:pKSE401-gPLL2:3rd gRNA
1250-Sl06g076100DT3-BsR	Solyc06g076100	ATTATTGGTCTCGAAAC**TTCAGCTGTTCCTTCAATC**c	PvuII	CRISPR:pKSE401-gPLL2:3rd gRNA
Sl06g076100-182	Solyc06g076100	CCACAACTTTCCGGTCAATCTC		Genotyping:*slpll2* ;1st gRNA
Sl06g076100-680R	Solyc06g076100	CCCACAACCCAATCACTTTCT		Genotyping:*slpll2* ;1st gRNA
Sl06g076100-772	Solyc06g076100	GTTCAGTGGGCTCAAGGTAAAG		Genotyping:*slpll2* ;2nd and 3rd gRNA
Sl06g076100-1381R	Solyc06g076100	CAGGGTTCTCCATATTCATCCTATC		Genotyping:*slpll2* ;2nd and 3rd gRNA
U6-29p-F		TTAATCCAAACTACTGCAGCCTGAC		3 sgRNA CRISPR:pKSE401 construct: 3gRNA against SlPLL2
U6-1p-R		TATGCAAGTCTCACTCACACTCACG		3 sgRNA CRISPR:pKSE401 construct: 3gRNA against SlPLL2

**Table S5 - Primers for RT-qPCR**		
Primer name	Gene ID	Sequence (5'-3')
elf1α 1272	Solyc06g009960 (*ToEF1α*)	AGCCCATGGTTGTTGAGACCTTTG
elf1α 1461R	Solyc06g009960 (*ToEF1α*)	TTCGAAACACCAGCATCACACTGC
ubi3 230F	Solyc01g056940 (*Ubiquitin3*)	CTCTTGCCGACTACAACATCC
ubi3 451R	Solyc01g056940 (*Ubiquitin3*)	AGCACCGCACTCAGCATTA
β-tub 317F	Solyc04g081490 (*beta-Tubulin*)	TAGAGCCTGGTACGATGGATAG
β-tub 450R	Solyc04g081490 (*beta-Tubulin*)	CAACTCAGCGCCTTCAGTATAA
ACT-816F	Solyc03g078400 (*SlActin7*)	CACTACTGCTGAACGGGAAAT
ACT-951R	Solyc03g078400 (*SlActin7*)	CTGTCCATCTGGCAACTCATAG
SlPI2-161	Solyc03g020080 (*SlPI-II*)	GGATATGCCCACGTTCAGAAGGAA
SlPI2-418R	Solyc03g020080 (*SlPI-II*)	AATAGCAACCCTTGTACCCTGTGC
PLL5-1776	Solyc06g076100 (*SlPLL2*)	GTTCAGAATCGACTATATTGGAACTTCC
PLL5-1963R	Solyc06g076100 (*SlPLL2*)	CTGGATCTCCGTCTGGTGACC

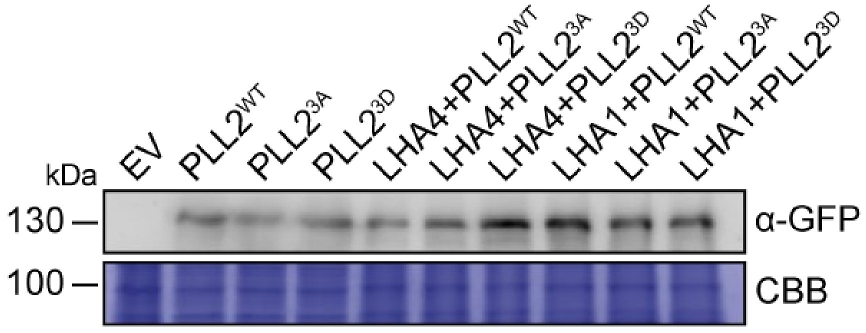

Supplementary Figure S1 | SlPLL2 variants are expressed at similar levels in yeast

SlPLL2 (PLL2^{WT}) and its phospho-mimetic (PLL2^{3D}) and phospho-dead (PLL2^{3A}) variants were expressed as GFP fusion proteins in yeast, either alone or together with the proton pumps LHA1 or LHA4. Empty-vector (EV)- transformants were used as negative control. Protein extracts were separated by SDS-PAGE and blotted to nitrocellulose. The western blot was developed with an anti-GFP serum. A duplicate Coomassie Brilliant Blue-stained gel is shown as loading control (CBB). The position of marker proteins is indicated and their mass is shown in kDa.

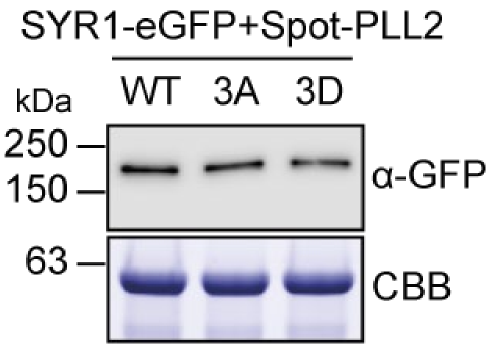

Supplementary Figure S2 | Transient expression of SYR1-eGFP to enable systemin perception in *N. benthamiana*

SYR1-GFP was co-expressed with Spot-tagged SlPLL2 (WT) and its phospho-mimetic (3D) and phospho-dead (3A) variants in *N. benthamiana* leaves by agro-infiltration. The ratio of agrobacteria carrying the different expression constructs was 5 (PLL2) : 3 (SYR1-GFP) : 2 (P19 suppressor of silencing). Protein extracts were separated by SDS-PAGE and blotted to nitrocellulose. The western blot was developed with an anti-GFP serum. A duplicate Coomassie Brilliant Blue-stained gel is shown as loading control (CBB). The position of marker proteins is indicated and their mass is shown in kDa.

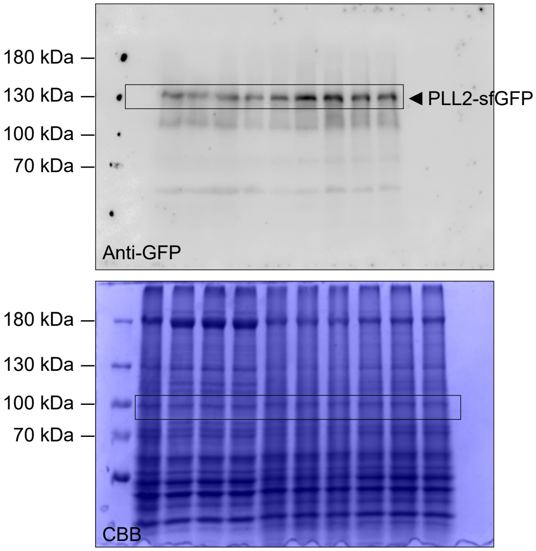

Supplementary Fig. S3 | Unprocessed gel and blot for Supplementary Fig. S1

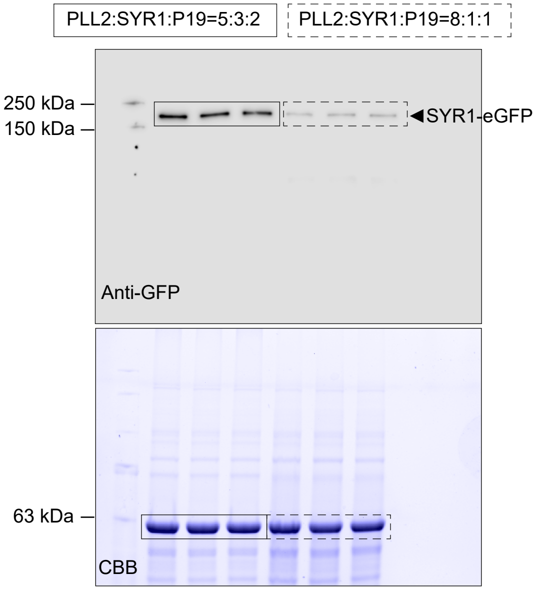

Supplementary Fig. S4 | Unprocessed gel and blot for Supplementary Fig. S2
