# Supplementary material for: Poltergeist-Like 2 (PLL2)-dependent activation of herbivore defence distinguishes systemin from other immune signalling pathways
Source: Nat Plants. 2025 Jul 4;11(7):1270–81. doi: 10.1038/s41477-025-02040-7 (PMC12283378; doi:10.1038/s41477-025-02040-7)
Supplement: Supplementary file 2 — Reporting Summary [file 41477_2025_2040_MOESM2_ESM.pdf]

nature portfolio

Corresponding author(s): Andreas Schaller
Annick Stintzi

Last updated by author(s): May 20, 2025

Reporting Summary

Nature Portfolio wishes to improve the reproducibility of the work that we publish. This form provides structure for consistency and transparency in reporting. For further information on Nature Portfolio policies, see our Editorial Policies and the Editorial Policy Checklist.

Statistics

For all statistical analyses, confirm that the following items are present in the figure legend, table legend, main text, or Methods section.

n/a	Confirmed	
☐	☒	The exact sample size (n) for each experimental group/condition, given as a discrete number and unit of measurement
☐	☒	A statement on whether measurements were taken from distinct samples or whether the same sample was measured repeatedly
☐	☒	The statistical test(s) used AND whether they are one- or two-sided<br>*Only common tests should be described solely by name; describe more complex techniques in the Methods section.*
☒	☐	A description of all covariates tested
☐	☒	A description of any assumptions or corrections, such as tests of normality and adjustment for multiple comparisons
☐	☒	A full description of the statistical parameters including central tendency (e.g. means) or other basic estimates (e.g. regression coefficient) AND variation (e.g. standard deviation) or associated estimates of uncertainty (e.g. confidence intervals)
☐	☒	For null hypothesis testing, the test statistic (e.g. F, t, r) with confidence intervals, effect sizes, degrees of freedom and P value noted<br>*Give P values as exact values whenever suitable.*
☒	☐	For Bayesian analysis, information on the choice of priors and Markov chain Monte Carlo settings
☒	☐	For hierarchical and complex designs, identification of the appropriate level for tests and full reporting of outcomes
☒	☐	Estimates of effect sizes (e.g. Cohen's d, Pearson's r), indicating how they were calculated

Our web collection on statistics for biologists contains articles on many of the points above.

Software and code

Policy information about availability of computer code

Data collection	CFX manager (BioRad) was used for the acquisition of qPCR data on a CFX Connect (BioRad) real-time PCR machine. Confocal imaging for BiFC and subcellular localization studies were performed on Zeiss LSM700 and LSM980 microscopes with ZEN black edition (2010) and Zen blue edition (version (v.) 3.6) microscopy software, respectively. Image Studio (v. 5.5) was used with a LICORbio Odyssey XF imager for chemiluminescence detection on western blots.
Data analysis	MaxQuant (v. 2.4.2.0) was used for mass spectrometry raw data analysis. R (v. 4.4.0) was used for linear regression (Figure 2a) and for the analysis of phosphoproteomics data. Specific R packages included limma (3.60.4), ggplot2 (3.5.1), tidyverse (2.0.0), missForest (1.5), stringr (1.5.1), factoextra (1.0.7), reshape2 (1.4.4), hypeR (2.2.0), ggpubr (0.6.0); viridis (0.6.5). R scripts are made available at GitHub (https://github.com/shibalili/systemin-project). qPCR data analysis was done with CFX Manager (BioRad). GraphPad Prism (v. 9.0) was used for statistical analyses and box plots, PhyloGenes v4.1 (www.phylogenes.org) based on GIGA algorithm (DOI:10.1186/1471-2105-11-312)) for phyolgenetic analyses, and Fiji (ImageJ v. 2.0.0/1.52p) for image analysis. Sequence data were analyzed with SnapGene (v. 7.0.2).

For manuscripts utilizing custom algorithms or software that are central to the research but not yet described in published literature, software must be made available to editors and reviewers. We strongly encourage code deposition in a community repository (e.g. GitHub). See the Nature Portfolio guidelines for submitting code & software for further information.

Data

Policy information about availability of data

All manuscripts must include a data availability statement. This statement should provide the following information, where applicable:

- Accession codes, unique identifiers, or web links for publicly available datasets
- A description of any restrictions on data availability
- For clinical datasets or third party data, please ensure that the statement adheres to our policy

Solanum lycopersicum genome annotation ITAG3.2 from PHYTOZOME V13.0 (https://phytozome-next.jgi. doe.gov/) was used for phospho-site mapping. MapMan ontology from MapManstore (https://mapman.gabipd.org/mapmanstore) was used for functional annotation and over-representation analysis. The mass spectrometry proteomics data have been deposited to the ProteomeXchange Consortium via the PRIDE partner repository with dataset identifiers PXD054229 and PXD062081. Raw data are included with the manuscript as source data, and are available with doi:10.6084/m9.figshare.28175321 at figshare.

Research involving human participants, their data, or biological material

Policy information about studies with human participants or human data. See also policy information about sex, gender (identity/presentation), and sexual orientation and race, ethnicity and racism.

Reporting on sex and gender	na
Reporting on race, ethnicity, or other socially relevant groupings	na
Population characteristics	na
Recruitment	na
Ethics oversight	na

Note that full information on the approval of the study protocol must also be provided in the manuscript.

Field-specific reporting

Please select the one below that is the best fit for your research. If you are not sure, read the appropriate sections before making your selection.

☒ Life sciences ☐ Behavioural & social sciences ☐ Ecological, evolutionary & environmental sciences

For a reference copy of the document with all sections, see nature.com/documents/nr-reporting-summary-flat.pdf

Life sciences study design

All studies must disclose on these points even when the disclosure is negative.

Sample size	Sample size was chosen following the example in previously published studies, see e.g. doi:10.1074/mcp.RA119.001367 for phospho proteomics, doi:10.1186/s12870-014-0257-8 for insect feeding assays, and doi:10.1016/j.devcel.2024.11.005 for root growth assays. In other cases, like ROS burst analysis (doi10.1007/978-1-0716-3511-7_12) and alkalinization response (doi:10.1007/978-1-0716-3511-7_13), the choice of sample size was based on our own experience to offer sufficient statistical power.
Data exclusions	In phosphoproteomics data analysis, reverse sequences, potential contaminants, and phosphosites with localization probability ≤ 0.75 were excluded due to poor mapping or low PTM confidence. For K-means clustering, phosphosites with ≤10 measurements were excluded because of their comparatively poor time profiles. One obvious outlier was removed manually from the data shown in Fig. 3n.
Replication	The experimental findings were reliably reproduced. Replication is reported for each experiment in the methods section or in the figure legends. Data for replicated experiments are included as 'source data'.
Randomization	Plants were randomly picked for the control and experimental groups to obtain similar numbers of individuals in each group.
Blinding	No randomized controlled trials were conducted, hence blinding was not generally applied. However, samples were anonymized for protein, DNA, and RNA extraction, phospho-peptide enrichment and trypsin digestion.

Reporting for specific materials, systems and methods

We require information from authors about some types of materials, experimental systems and methods used in many studies. Here, indicate whether each material, system or method listed is relevant to your study. If you are not sure if a list item applies to your research, read the appropriate section before selecting a response.

Materials & experimental systems

n/a	Involved in the study
☐	☒ Antibodies
☐	☒ Eukaryotic cell lines
☒	☐ Palaeontology and archaeology
☒	☐ Animals and other organisms
☒	☐ Clinical data
☒	☐ Dual use research of concern
☐	☒ Plants

Methods

n/a	Involved in the study
☒	☐ ChIP-seq
☒	☐ Flow cytometry
☒	☐ MRI-based neuroimaging

Antibodies

Antibodies used

The following antibodies were used in Western blot analyses: anti-GFP (1:10000, Sigma-Aldrich, #SAB4301138); anti-Flag-HRP (1:5000, Sigma-Aldrich, A8592); anti-pERK1/2 (1:5000; Phospho-p44/42 MAPK (Erk1/2) (Thr202/Tyr204) (D13.14.4E) XP® Rabbit mAb, #4370, Cell Signaling Technology); goat anti-rabbit IgG-HRP (1:10000; Invitrogen, #31460); goat anti-mouse IgG-HRP (1:10000; Sigma-Aldrich, #DC02L); anti-RGS-6x-His (1:1000; Qiagen, #34650). The polyclonal antiserum against AtAHA2 was generated by Serrano et al., 1991 (PMID: 1834646). It was custom-made by Genofit (Geneva, Switzerland) using the C-terminus of AtAHA2 (residues 851-949) expressed in E. coli as the antigen.

Validation

For the AtAHA2 antiserum, specific recognition of plasma membrane proton pumps from various plant species was confirmed by Serrano et al., 1991 (PMID: 1834646). For the commercial antibodies, details of the validation procedures are given on the manufacturer's websites: Affinity purified anti-GFP was validated for western blotting at 1:1000-1:10000, monoclonal anti-Flag-HRP was validated for dot blotting with a detection limit of 32 ng at 1:1000, anti-pERK1/2 was validated for western blotting using human, mouse, and rat cell lines, and anti-RGS-6xHis was validated for blotting with various recombinant proteins and negligible cross reactivity with crude E. coli, yeast, mammalian or insect cell lysates.

Eukaryotic cell lines

Policy information about cell lines and Sex and Gender in Research

Cell line source(s)

The Solanum perivianum cell suspension culture was obtained from Georg Felix (University of Tübingen). The cell line was first described by Nover et al., 1982 (https://doi.org/10.1016/S0015-3796(82)80041-3).

Authentication

PCR amplification and sequencing of the SYR1 locus.

Mycoplasma contamination

This is a plant cell line, and as such, it was not tested for mycoplasma contamination.

Commonly misidentified lines (See ICLAC register)

na

Dual use research of concern

Policy information about dual use research of concern

Hazards

Could the accidental, deliberate or reckless misuse of agents or technologies generated in the work, or the application of information presented in the manuscript, pose a threat to:

No	Yes
☒	☐ Public health
☒	☐ National security
☒	☐ Crops and/or livestock
☒	☐ Ecosystems
☒	☐ Any other significant area

Experiments of concern

Does the work involve any of these experiments of concern:

No	Yes	
☒	☐	Demonstrate how to render a vaccine ineffective
☒	☐	Confer resistance to therapeutically useful antibiotics or antiviral agents
☒	☐	Enhance the virulence of a pathogen or render a nonpathogen virulent
☒	☐	Increase transmissibility of a pathogen
☒	☐	Alter the host range of a pathogen
☒	☐	Enable evasion of diagnostic/detection modalities
☒	☐	Enable the weaponization of a biological agent or toxin
☒	☐	Any other potentially harmful combination of experiments and agents

Plants

Seed stocks	Solanum lycopersicum cv UC82B was used as the tomato wild-type genotype. Seeds were obtained form Starke Ayres, South Africa (https://www.starkeayres.com/home). Nicotiana benthamiana seeds were obtained from Agroscience GmbH (Neustadt, Germany).
Novel plant genotypes	CRISPRdirect (https://crispr.dbcls.jp) was used to select sgRNA target sites (3 for PLL2: GTTCCGGCTTCGGGCCCTA, CCATCTCCGAGTTTGGCCG, TTCAGCTGTTCCTTCAATC, 2 for SYR1 (CGGTAACTTGCCGGTCAAT, CGTTTGGTCTCCCTTGATC). Transgenic tomato plants were gerated by agrobacterium-mediated transformation of etiolated cotyledon explants as described (PMID25073705). The transgenic S. peruvianum cell culture was generated by particle bombardment as described (PMID: 19332543).
Authentication	Mutants were genotyped by PCR-amplification of the respective genomic loci and sequencing. syr1 and slpll2 mutant plants were identified among the primary tranformants (T0). Homozygous mutants from the segregating Cas9-free T2 progeny were used for experiments. Three independent mutant lines were analyzed to exclude off-target effects. In the syr1 mutant cell line, specific loss of SYR1 function was confirmed by comparing different elicitors of the alkalinization response. The response to systemin was impaired, while responses to flg22 and chitin remained unaffected. Off target effects at sites unrelated to the alkalinization response cannot be fully excluded.
