# Supplementary material for: Poltergeist-Like 2 (PLL2)-dependent activation of herbivore defence distinguishes systemin from other immune signalling pathways
Source: Nat Plants. 2025 Jul 4;11(7):1270–81. doi: 10.1038/s41477-025-02040-7 (PMC12283378; doi:10.1038/s41477-025-02040-7)

Figure 2c
Replicate 3

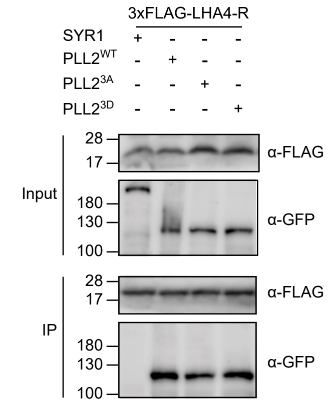

Replicate 1

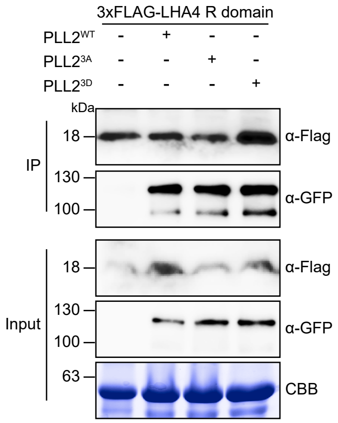

Replicate 2

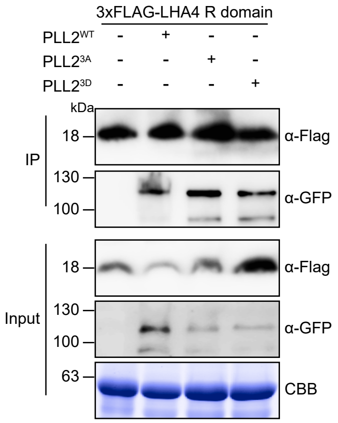

unprocessed western blots/gels
Replicate 3

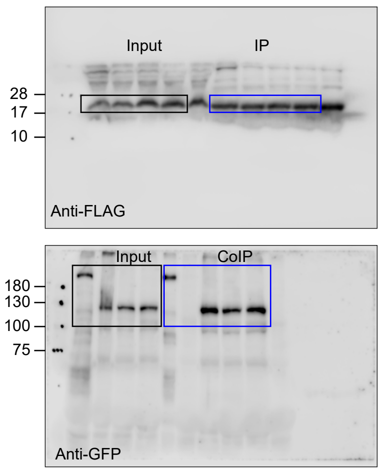

Replicate 1

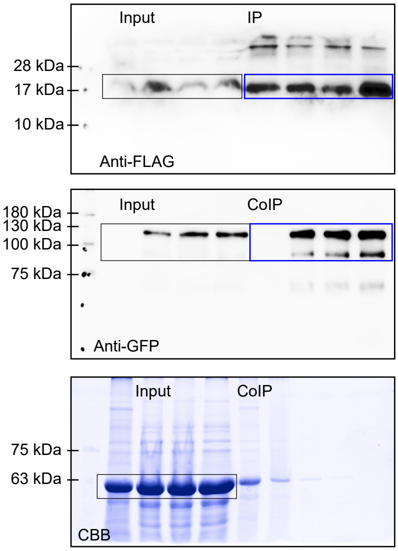

Replicate 2

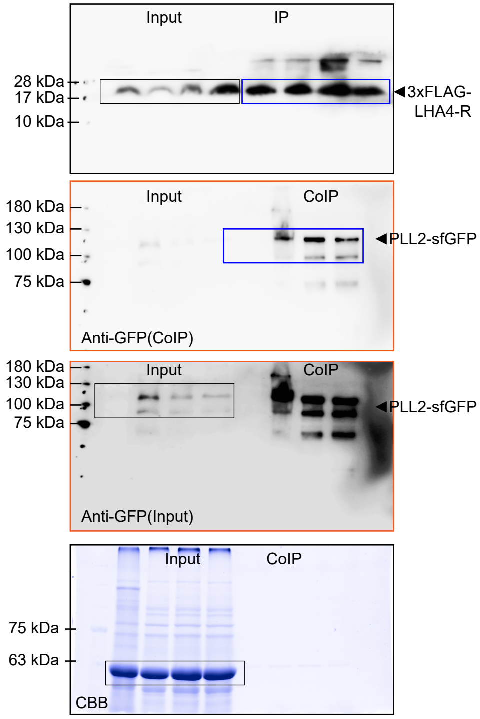

Figure 2d

replicate 1

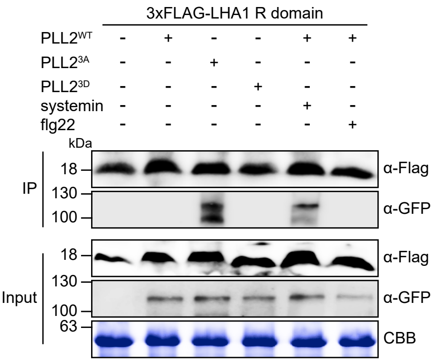

replicate 2 (shown as Fig. 2d)

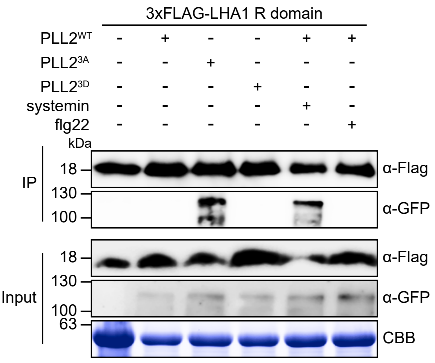

unprocessed western blots/gels

replicate 1

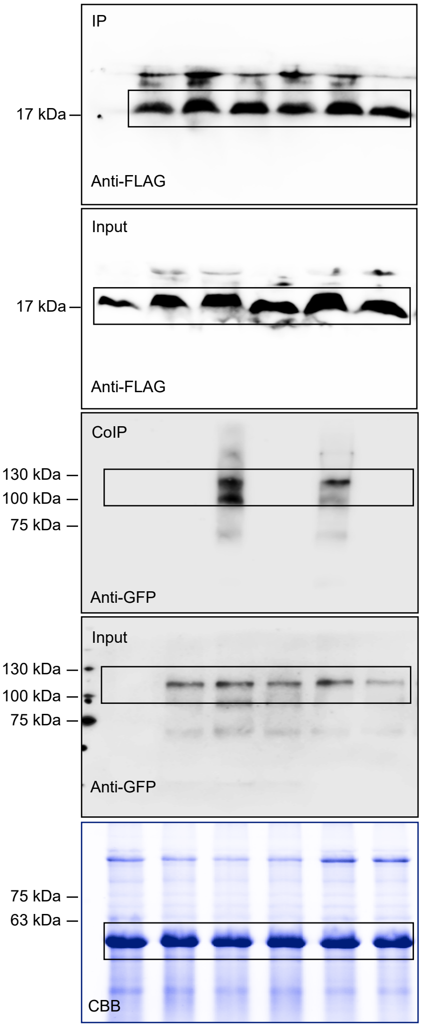

replicate 2 (shown as Fig. 2d)

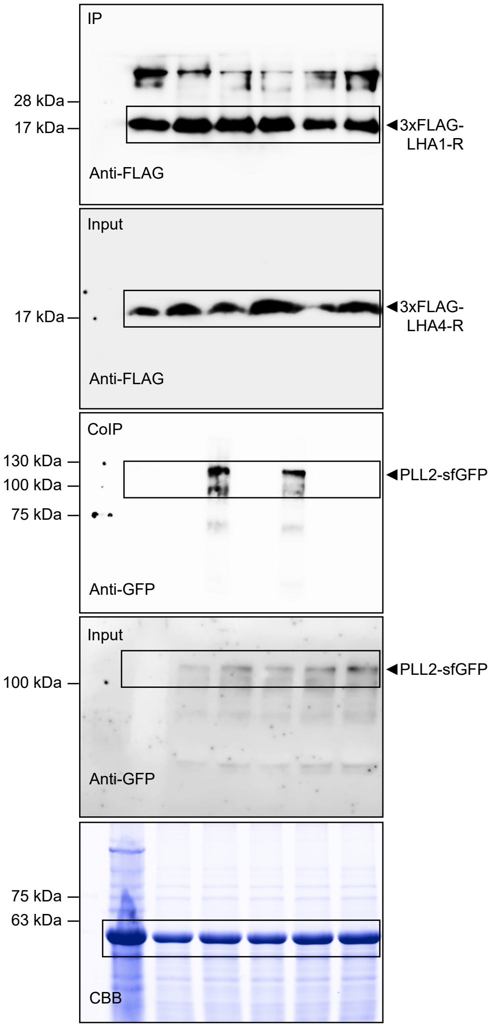

Figure 2e

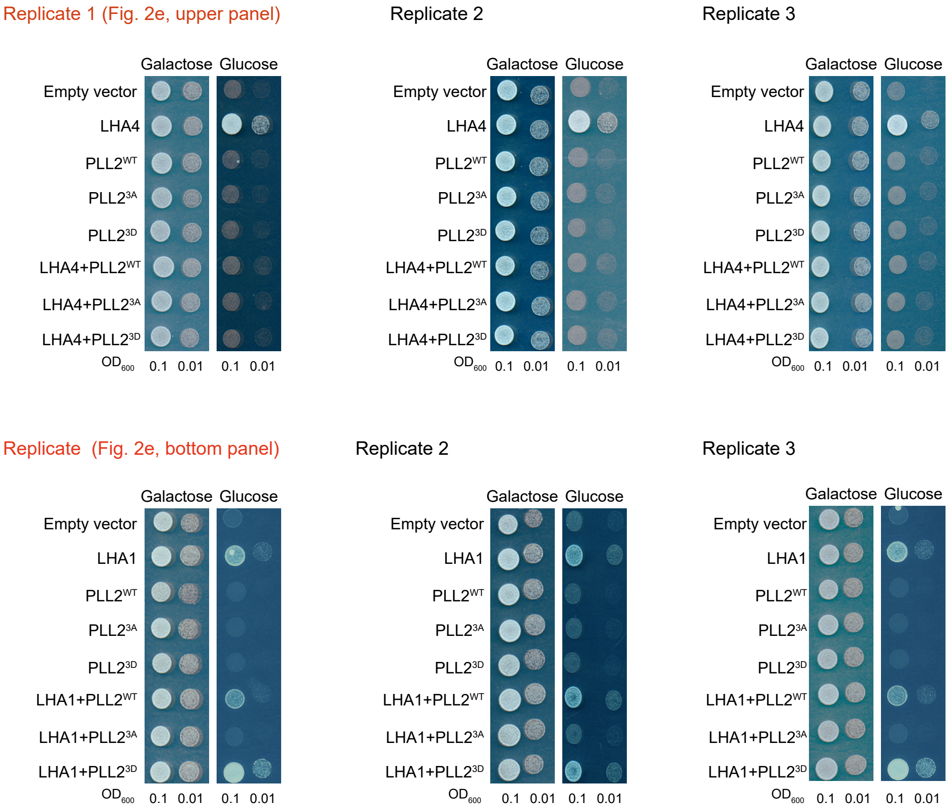

Replicate 1 (Fig. 2e, upper panel)
Replicate 2
Replicate 3
Galactose
Glucose
Empty vector
LHA4
PLL2^{WT}
PLL2^{3A}
PLL2^{3D}
LHA4+PLL2^{WT}
LHA4+PLL2^{3A}
LHA4+PLL2^{3D}
OD$_{600}$
0.1
0.01
Replicate (Fig. 2e, bottom panel)
Replicate 2
Replicate 3
Galactose
Glucose
Empty vector
LHA1
PLL2^{WT}
PLL2^{3A}
PLL2^{3D}
LHA1+PLL2^{WT}
LHA1+PLL2^{3A}
LHA1+PLL2^{3D}
OD$_{600}$
0.1
0.01

Figure 2f, upper panel

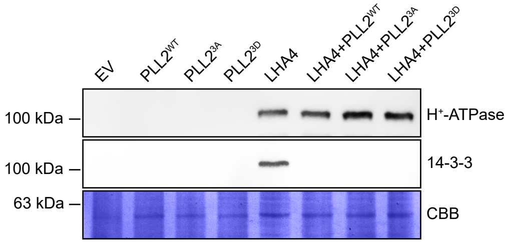

unprocessed western blots/gels

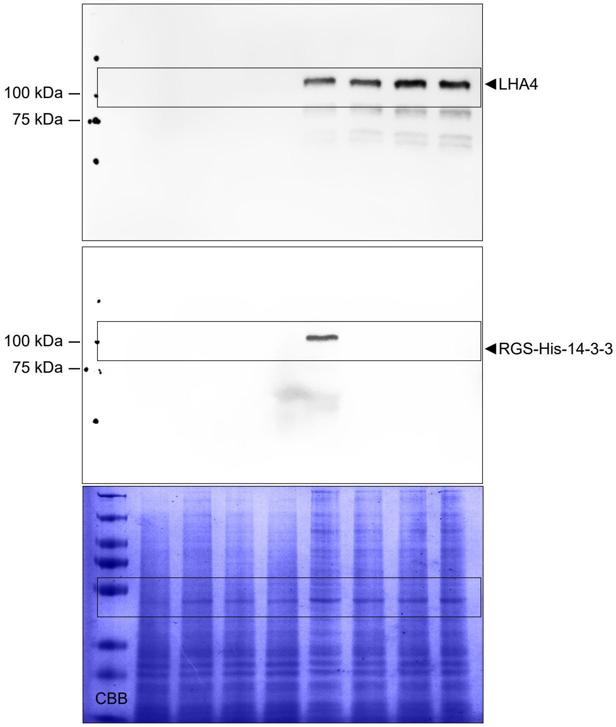

Figure 2f, bottom panel

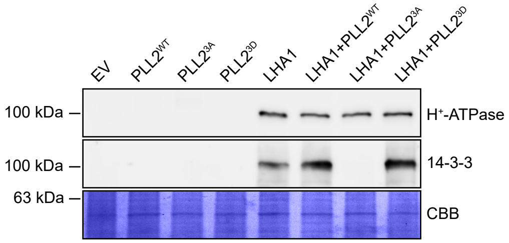

unprocessed western blots/gels

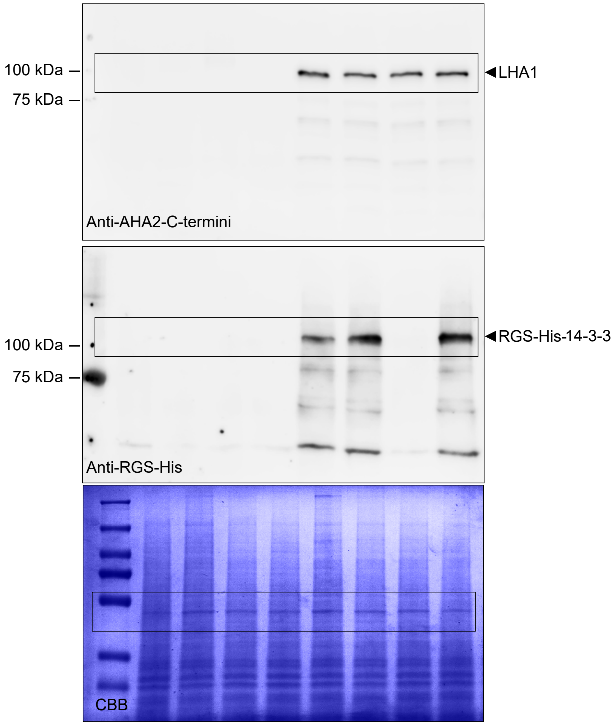

Supplement: Supplementary file 5 — Unprocessed western blots and/or gels with replicates. [file 41477_2025_2040_MOESM5_ESM.pdf]
