# Supplementary material for: Poltergeist-Like 2 (PLL2)-dependent activation of herbivore defence distinguishes systemin from other immune signalling pathways
Source: Nat Plants. 2025 Jul 4;11(7):1270–81. doi: 10.1038/s41477-025-02040-7 (PMC12283378; doi:10.1038/s41477-025-02040-7)

Figure 3a

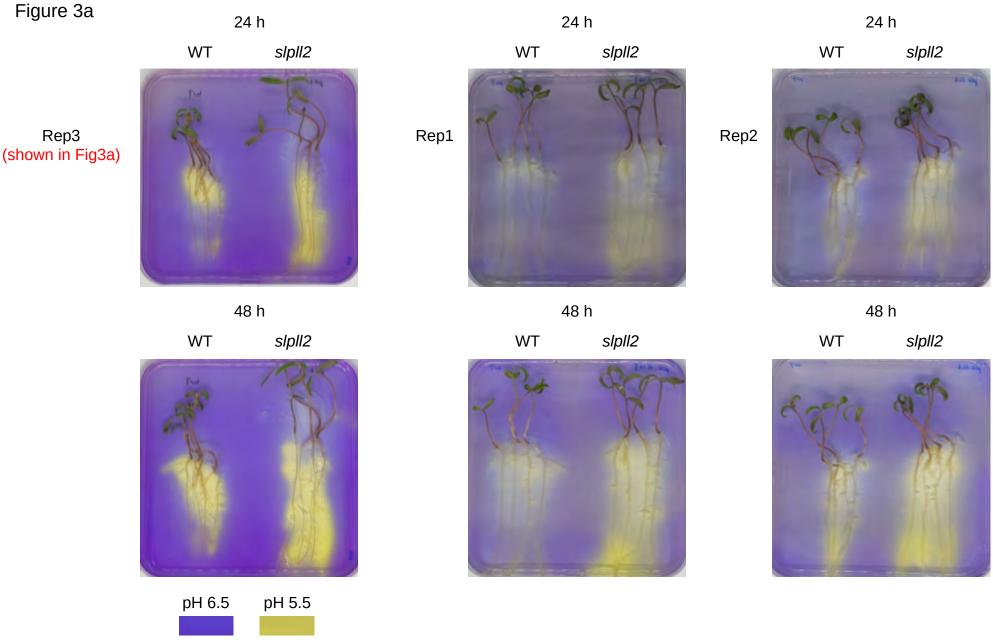

Figure 3b

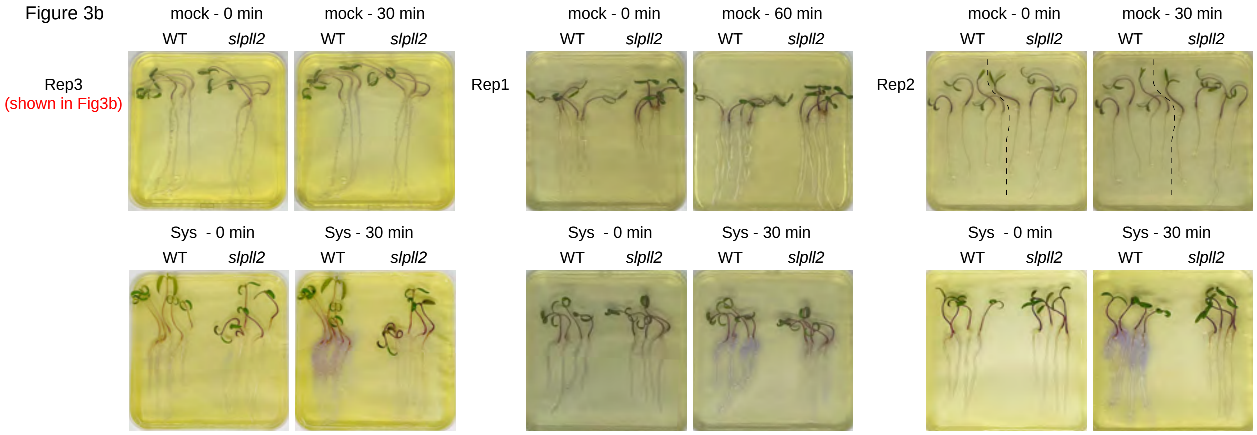

Figure 3e

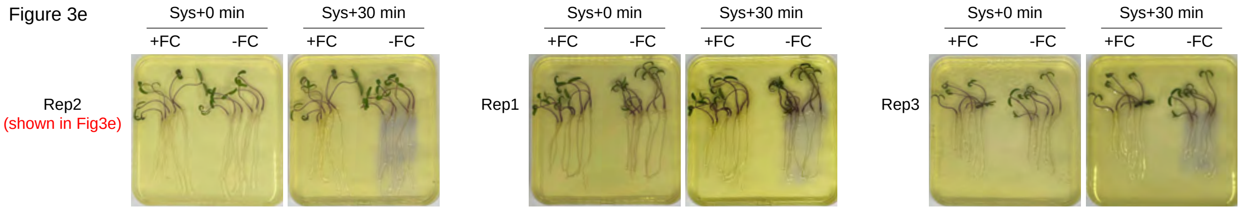

Figure 3f

Replicate 1 (shown as Fig. 3f)

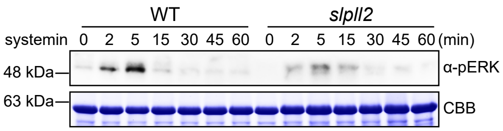

unprocessed western blots/gels: Replicate 1

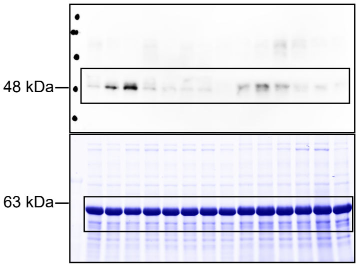

Replicate 2

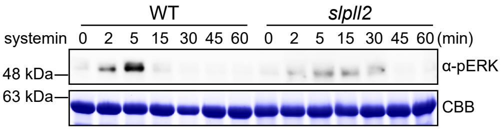

unprocessed western blots/gels: Replicate 2

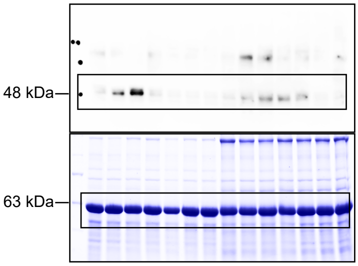

Supplement: Supplementary file 7 — Unprocessed western blots and/or gels with replicates. [file 41477_2025_2040_MOESM7_ESM.pdf]
