# Supplementary figures and images for: Poltergeist-Like 2 (PLL2)-dependent activation of herbivore defence distinguishes systemin from other immune signalling pathways
Source: Nat Plants. 2025 Jul 4;11(7):1270–81. doi: 10.1038/s41477-025-02040-7 (PMC12283378; doi:10.1038/s41477-025-02040-7)

**A**

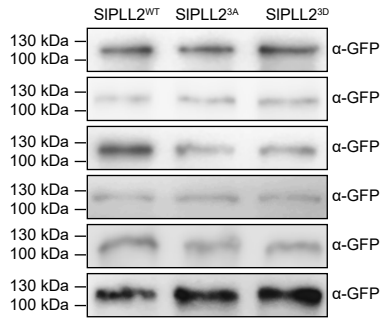

unprocessed western blots

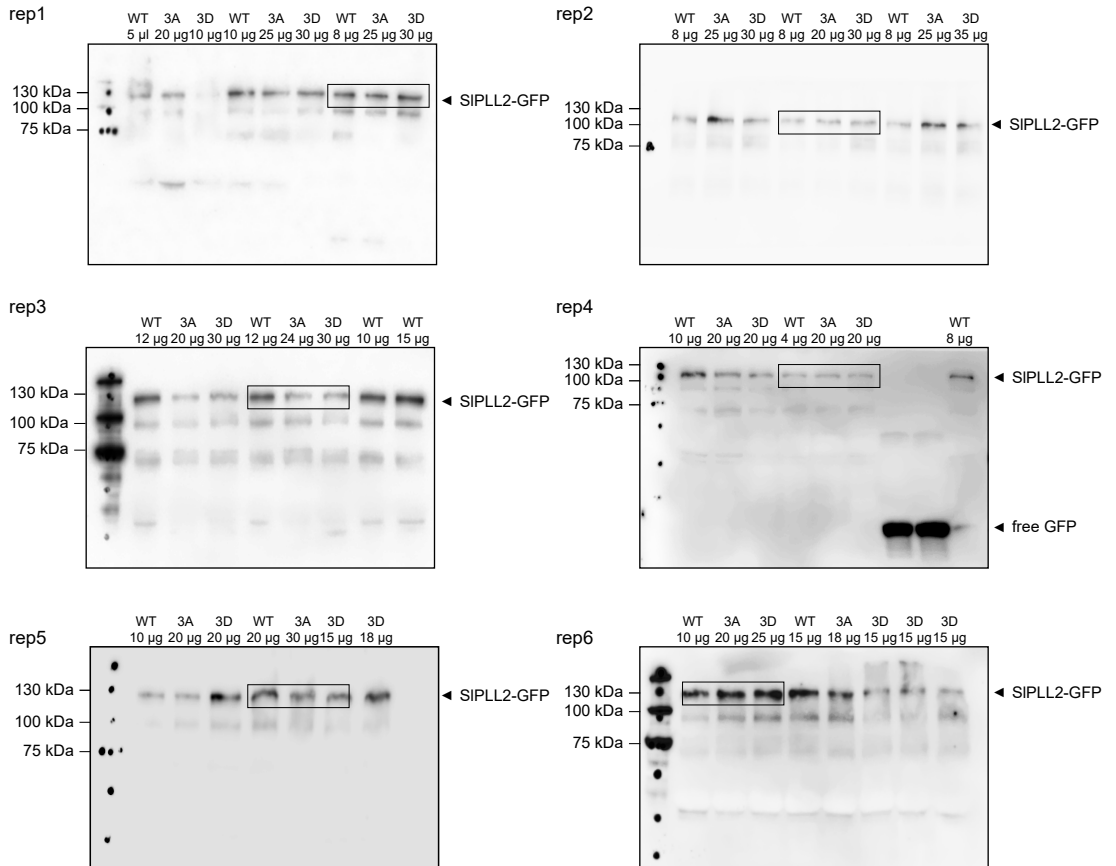

Supplement: Supplementary file 9 — Unprocessed western blots and/or gels with replicates. [file 41477_2025_2040_MOESM9_ESM.pdf]

Replicate 1 (shown as Extended Fig. 8b)

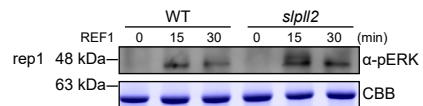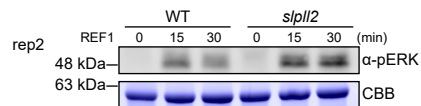

unprocessed western blots and gels

rep1

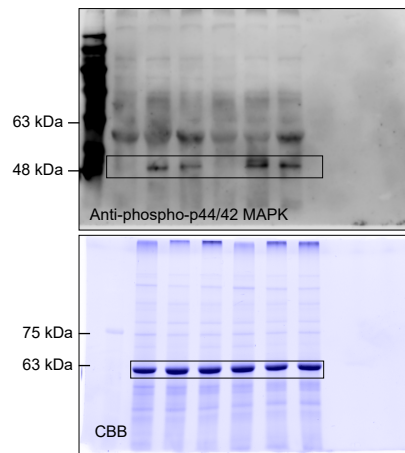

rep2

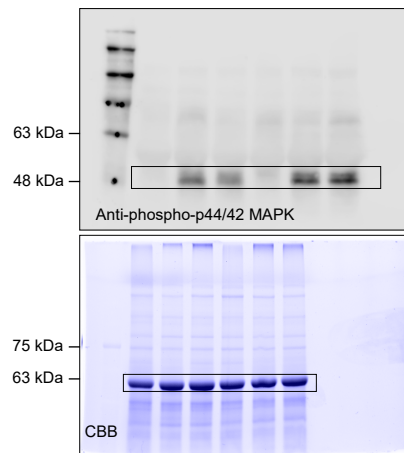

Supplement: Supplementary file 11 — Unprocessed western blots and/or gels with replicates. [file 41477_2025_2040_MOESM11_ESM.pdf]
